# A modified iChip for *in situ* cultivation of bacteria in arid environments

**DOI:** 10.1128/aem.01325-24

**Published:** 2025-01-08

**Authors:** Seven Nazipi Bushi, Marie B. Lund, Tobias Sandfeld, Sanne Sadolin Nørskov, Simon Fruergaard, Marianne Glasius, Trine Bilde, Andreas Schramm

**Affiliations:** 1 Department of Biology – Section for Microbiology, Aarhus University 683568 , Aarhus, Denmark; 2 Department of Chemistry, Aarhus University 114237 , Aarhus, Denmark; 3 Department of Biology – Section for Genetics, Ecology and Evolution, Aarhus University 683568 , Aarhus, Denmark; Universidad de los Andes, Bogotá, Colombia

**Keywords:** iChip, *in situ *cultivation, culturability, bacterial diversity, spider nest microbiome, volatile organic compounds

## Abstract

Antimicrobial resistance is an ever-increasing problem for human health, and with only a few novel antimicrobials discovered in recent decades, an extraordinary effort is needed to circumvent this crisis. A promising source of new microbial-derived antimicrobial compounds resides in the large fraction of microbes that are not readily cultured by standard cultivation. It has previously been shown that nests of the social spider *Stegodyphus dumicola* contain a diverse bacterial community, where only a small fraction of the microbes could be recovered by standard cultivation. To improve the recovery of the bacterial diversity cultured from nests, we modified the previously described isolation chip (iChip) to fit the natural arid environment of *S. dumicola* nests. Here we provide a comprehensive analysis of the modified iChip’s performance. We found that the modified iChip improved the overall culturability, performed equally or better at recovering the bacterial diversity from individual nests, and improved the recovery of rare isolates compared to standard cultivation. Furthermore, we show that the modified iChip can be used in the field. In addition, we observed that the nests contain volatile organic compounds (VOCs) that could serve as substrate for the selective enrichment of rare and iChip-specific isolates. Our modified iChip can be applied for *in situ* cultivation in a broad range of arid habitats that can be exploited for future drug discovery.

**IMPORTANCE:**

The demand for novel antimicrobial compounds is an ever-increasing problem due to the rapid spread of antibiotic-resistant microbes. Therefore, exploring new habitats for microbial-derived antimicrobial compounds is crucial. The nest microbiome of *Stegodyphus dumicola* remains largely unexplored and could potentially serve as a new source of antimicrobial compounds. To access the nest’s microbial diversity, we designed a modified iChip for *in situ* cultivation inside spider nests and tested its applications in both field and laboratory settings. Our study shows that the iChip’s ability to recover *in situ* abundant genera was comparable or superior to standard cultivation, while the recovery of rare (low-abundant genera) was higher. We argue that these low-abundant and iChip-specific isolates are enriched from naturally occurring nest volatile organic compounds (VOCs) during iChip incubation.

## INTRODUCTION

In light of the rapid development and spread of antibiotic resistance in bacteria, there is a renewed interest in finding novel antimicrobials (1–3). A promising source of new antimicrobial compounds may be found in yet-uncultured bacteria, and hence several new approaches have been developed over the past 20 years to improve the cultivation and isolation of previously uncultured microbial taxa (4–6). Many of these cultivation technologies aim at mimicking the microbes’ natural environment and growth conditions to improve their culturability (7–11). The isolation chip (iChip) is an example of an *in situ* cultivation device that has proven successful for the isolation of novel bacterial species and, intriguingly, for the discovery of an entirely new class of antibiotics (12, 13).

The original iChip is composed of several hundred through-holes that essentially function as miniaturized diffusion chambers in a central plate, each inoculated with a single microbial cell. To load (on average) a single cell into each diffusion chamber, the plate is immersed into a highly diluted agar suspension of the environmental inoculum. The plate is then sandwiched between membranes with a pore size of 0.03 µm and two outer compartments that allow the diffusion of small molecules while preventing contamination with cells from outside the diffusion chambers. The loaded and assembled iChip is then placed back into the environment (e.g., soil or sediment), and the cells inside the chambers eventually grow into visible microcolonies due to the diffusion of nutrients and growth factors from their natural environment (12). Since the introduction of the first iChip in 2010, Epstein and colleagues have simplified the design so that the device can be constructed using common laboratory materials, such as pipette tip racks (14), and it has also been modified for application in hot environments (15).

**Fig 1:**
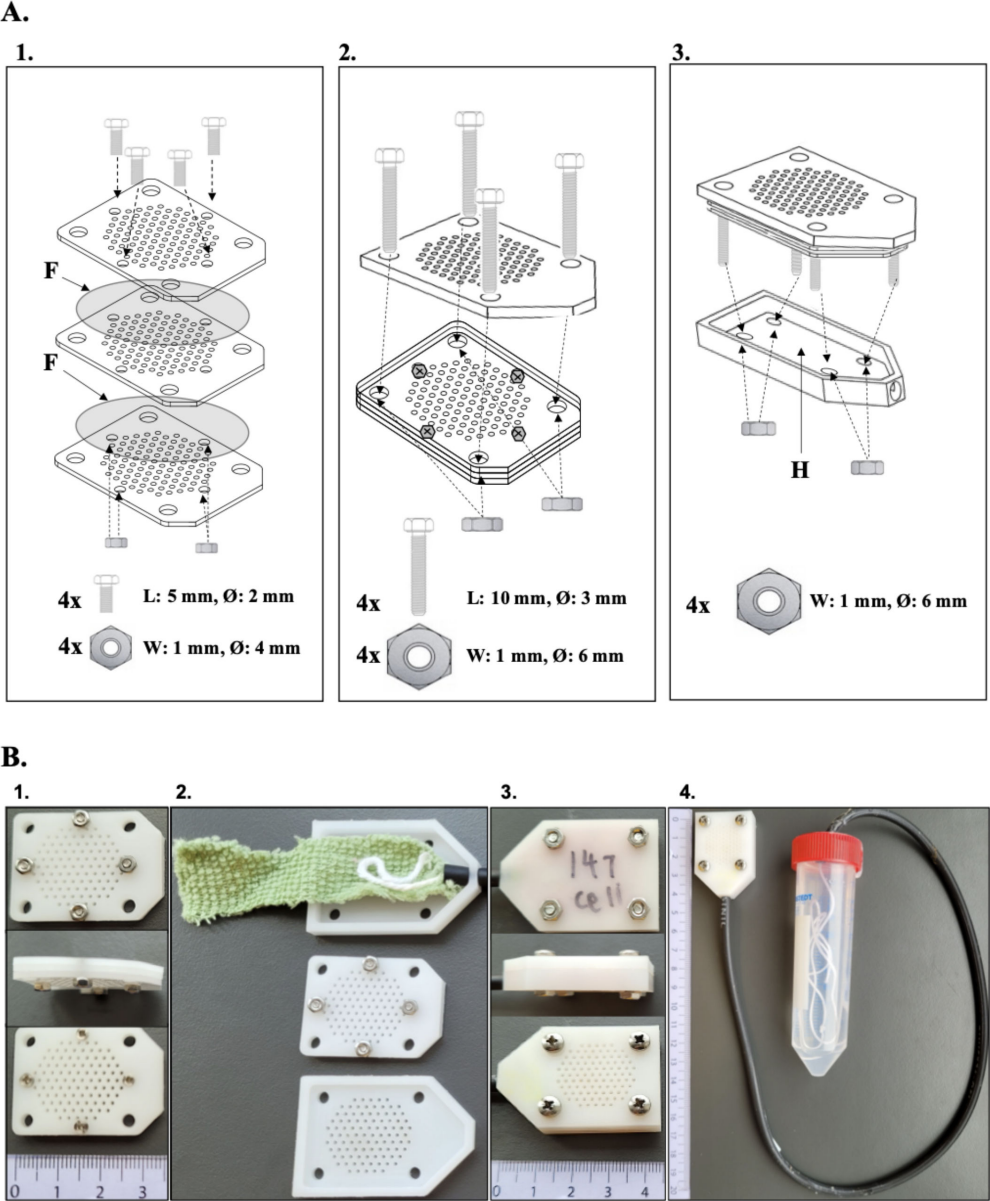
The modified iChip. (**A**) Schematic and assembly of the iChip. (1) The innermost plate is inoculated and sandwiched between two 0.03 µm filter membranes (F) and two additional plates. Four bolts and nuts are used to hold this central growth compartment together. (2) The central growth compartment is then attached to the outer plate by four larger bolts and nuts, and (3) finally attached to the modified bottom plate, acting as a hydration chamber (H), with four additional nuts. (**B**) The modified iChip in pictures. (1) Front view, side view, and back view of the assembled central growth compartment. (2) Bottom, central, and outer iChip compartment. The bottom plate is equipped with absorbing material, and the cotton wick is protruding from the black ISO-VERSINIC tube. (3) Back view, side view, and front view of the assembled iChip. (4) Fully assembled iChip connected to the water reservoir via an ISO-VERSINIC tube.

Even though these iChips show promising results, their application is restricted to water-saturated environments or requires continued re-wetting (e.g., in soil), due to the rapid dehydration of the gelling agent found inside the central plate of the iChip (14). To overcome the dehydration problem and thus expand its application to arid environments, we adapted the previously described iChip to arid environments by adding a water reservoir that enables a continuous flow of water into the iChip by capillary forces, which keeps the gelling-agent hydrated (Fig. 1). In addition, our modified iChip is miniaturized for the application inside nests of African social spiders (*Stegodyohus dumicola*).

These social spiders have extremely low species-wide genetic diversity which in combination with group living increases the risk of pathogen transmission among nest mates (16–18). It was therefore hypothesized that antimicrobial compounds produced by spider- and nest-associated microbes play a role in host protection against pathogens (19, 20). Social spiders build tunneled communal nests composed of various organic materials (invertebrate prey carcasses, branches, and leaves) entangled in a very dense layer of spider silk (21–24). These nests are inhabited by a diverse fungal and bacterial community, of which so far only a tiny fraction but none of its core microbiome members has been cultured using conventional cultivation methods (19), which makes the nests an ideal place to test the iChip’s ability to improve the cultured diversity.

Due to dry conditions inside the nests, we expected that the primary nutrients available to microbes in the nest were volatile organic compounds (VOCs) that could diffuse through the tunneled nest passages and would also enter the iChip diffusion chambers. *Stegodyphus dumicola* nests indeed were shown to contain a complex volatilome with a mix of animal, plant, and microbial VOCs (20) some even with antimicrobial activity (25).

The goal of our study was to modify the iChip for *in situ* cultivation under dry conditions, specifically for use inside social spider nests. We evaluated its performance with laboratory spider colonies by comparing the iChip-recovered bacterial isolates to (i) the bacterial community in the nest (analyzed by 16S rRNA gene amplicon sequencing) and (ii) the bacterial isolates recovered by standard cultivation on agar plates. In addition, we analyzed the laboratory nests for VOCs, which might have served as substrates for microcolony growth inside the iChips. Finally, we tested the field applicability by deploying iChips in African *S. dumicola* nests in Otavi (Namibia) to test whether the modified iChip could withstand dehydration under real-life extreme dry and hot conditions.

## RESULTS AND DISCUSSION

### Modification of the iChip for use under hot and dry conditions in spider nests

The primary modification of the iChip is the addition of a hydration chamber, which consists of an absorbing material (micro-fiber cloth) connected to a water reservoir *via* a cotton thread inside an ISO-VERSINC tube (Fig. 1). The ISO-VERSINC tube is highly temperature resistant, which hinders evaporation during water transport through the cotton thread. As water evaporates from inside the iChip, capillary forces will draw in water from the reservoir through the absorbing microfiber cloth and the cotton thread, keeping the central growth compartment hydrated. A second modification is the miniaturization of the iChip (43 × 29 × 8 mm) for application inside spider nests, which have typically diameters below 25 cm (26). The modified iChip contains 100 through-holes as cultivation chambers (diameter, 1 mm; depth, 1 mm) arranged in a circular pattern that can be covered by a sterile filter membrane (diameter, 25 mm); see Fig. 1; Fig. S1 for details.

### Experimental design of the laboratory test

**Fig 2:**
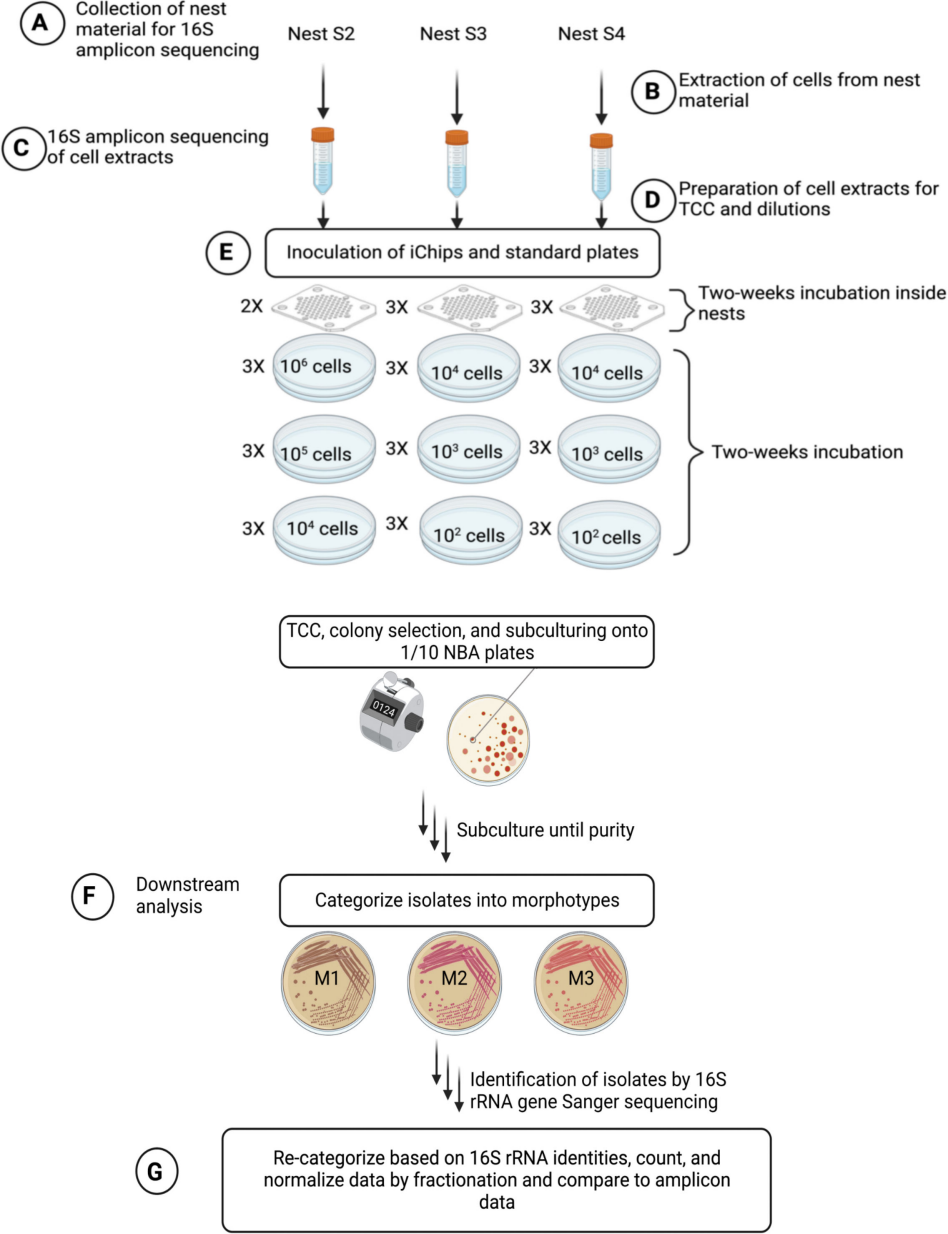
Workflow of the laboratory test. (**A**) Nest material (~1.6 g) was sampled from three areas of each nest (*n* = 3) and pooled for 16S rRNA gene amplicon sequencing. (**B**) Cells were detached from nest material into phosphate-buffered saline (PBS) by brief sonication and vortexing. Larger particles were allowed to settle, and the resulting cell extracts were filtered through 0.8 µm filters and aliquoted. (**C**) 1 mL of aliquots of the cell extracts was used for 16S rRNA gene amplicon sequencing. (**D**) The remaining aliquots of each extract were diluted and either stained with DAPI for total cell counts (TCC) or further diluted for iChip and agar plate inoculation. (**E**) Triplicate iChips were inoculated and inserted into each nest except for nest S2, where one uninoculated iChip served as a negative control. Triplicate 1:10 NBA plates were inoculated with three different cell concentrations per nest. (**F**) After 2 weeks of *in situ* incubation, iChips were dismantled, their agar plugs dissolved in PBS, and transferred for subculturing onto 1:10 NBA plates. Colonies from both iChip-subculturing and standard 1:10 NBA plates were picked, sub-cultured until purity, and categorized into morphotypes. Representatives of each morphotype were identified by 16S rRNA gene sequencing. (**G**) Based on 16S rRNA results, some morphotypes were re-categorized. Isolates were counted, counts were normalized by fractionation, and cultivation data were compared to amplicon sequence data. The illustration was created with BioRender.com.

We evaluated the performance of the modified iChip using three spider nests kept in the laboratory under a temperature regime mimicking natural conditions (see Materials and Methods for details). In that way, handling and inoculation could be performed in a laminar flow bench, and a thorough analysis of cultivation biases and potential contamination was possible by comparing the iChip results with standard cultivation (colony-forming units [CFU] on dilute nutrient broth agar [NBA] 1:10, under the same temperature regime as the iChips) and cultivation-independent analyses (16S rRNA gene amplicon sequencing of both the original nest material and the cell extracts from which the iChips and the standard cultivation plates were inoculated). One iChip was loaded with a sterile medium and served as a negative control. Figure 2 gives a detailed overview of the workflow for the controlled laboratory test, and Fig. S2 shows the laboratory setup during the iChip incubation in a spider nest.

### Culturability and diversity of nest-associated bacteria

Total cell count (TCC) varied between nests, from 2.7 × 10^7^ to 4.5 × 10^8^ cells mL^−1^, while culturability (defined as CFU divided by TCC) on standard NBA plates was on average 2.4% ± 1.4% (Table S1). Thus, culturability on our plates was slightly higher or comparable to the culturability reported from environmental samples under standard laboratory conditions (27). The culturability in the iChips could not be determined since the number of colonies subcultured from the iChips on 1:10 NBA plates surpassed the number of cells originally seeded into the iChips (Table S2). This is an unavoidable consequence of transferring whole agar plugs from the iChips into phosphate-buffered saline (PBS) for subculturing: the tiny size of the agar plugs lets them dry out very quickly as soon as the iChip is opened, and therefore the picking of individual microcolonies from agar plugs, as done in other iChip studies (14), was impossible. When dissolved in PBS, a single microcolony results in many new colonies on the inoculated plate, preventing exact quantification. However, if we assume that each of the 100 holes of the iChips on average contained a single cell (following a Poisson distribution) and that each phylotype (i.e., each isolates with a unique 16S rRNA sequence) (Table S2) originated from one microcolony, we get a culturability of 19%–29% as a conservative estimate, which is about 10 times better than with standard cultivation. The actual culturability in the iChips may have been even higher, as the same phylotype may represent several microcolonies.

**Fig 3:**
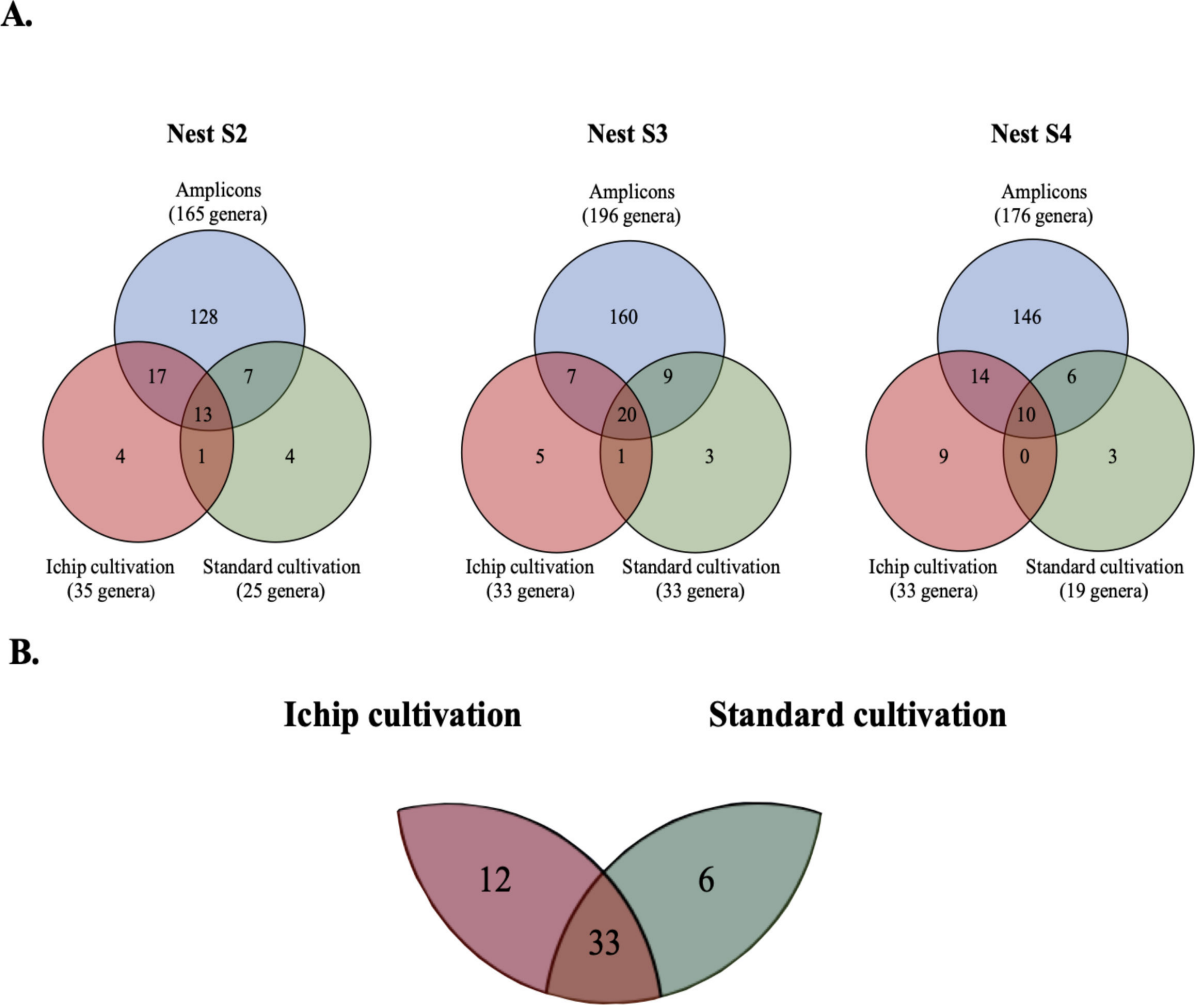
Comparison of molecular- and cultivation-based recovery of genus-level diversity from spider nests. (**A**) Venn diagrams showing the number of unique and shared genera between amplicon data, iChip, and standard cultivation, for each of the three nests. Amplicon data represent both nest material and cell extracts. Numbers in brackets show the total number of genera present in each of the circles. (**B**) Agglomeration of all genera that were detected by amplicon sequencing and recovered by iChip and standard cultivation from all three nests. See Table S4 for genus identification.

Amplicon sequencing of nest material and cell extracts (accumulated for the three nests) resulted in 1,118 amplified sequence variants (ASVs) clustered into 230 genera for the nest material, and 892 ASVs in 209 genera for the cell extracts (Table S3). By contrast, only 158 phylotypes in 62 genera were identified among all iChip isolates, and an even lower number (112 phylotypes in 48 genera) for the isolates from standard cultivation (Table S2). Detailed genus-level comparison for each nest (Fig. 3A) showed some variation between individual nests, but generally a trend for a higher representation of the 16S rRNA gene amplicon diversity by the iChip isolates (14%–18%) than by standard cultivation (9%–15%). Importantly, the iChip performed as good or better than standard cultivation to isolate representatives of the 25 most abundant microbiome genera in each nest (Fig. S3).

**Fig 4:**
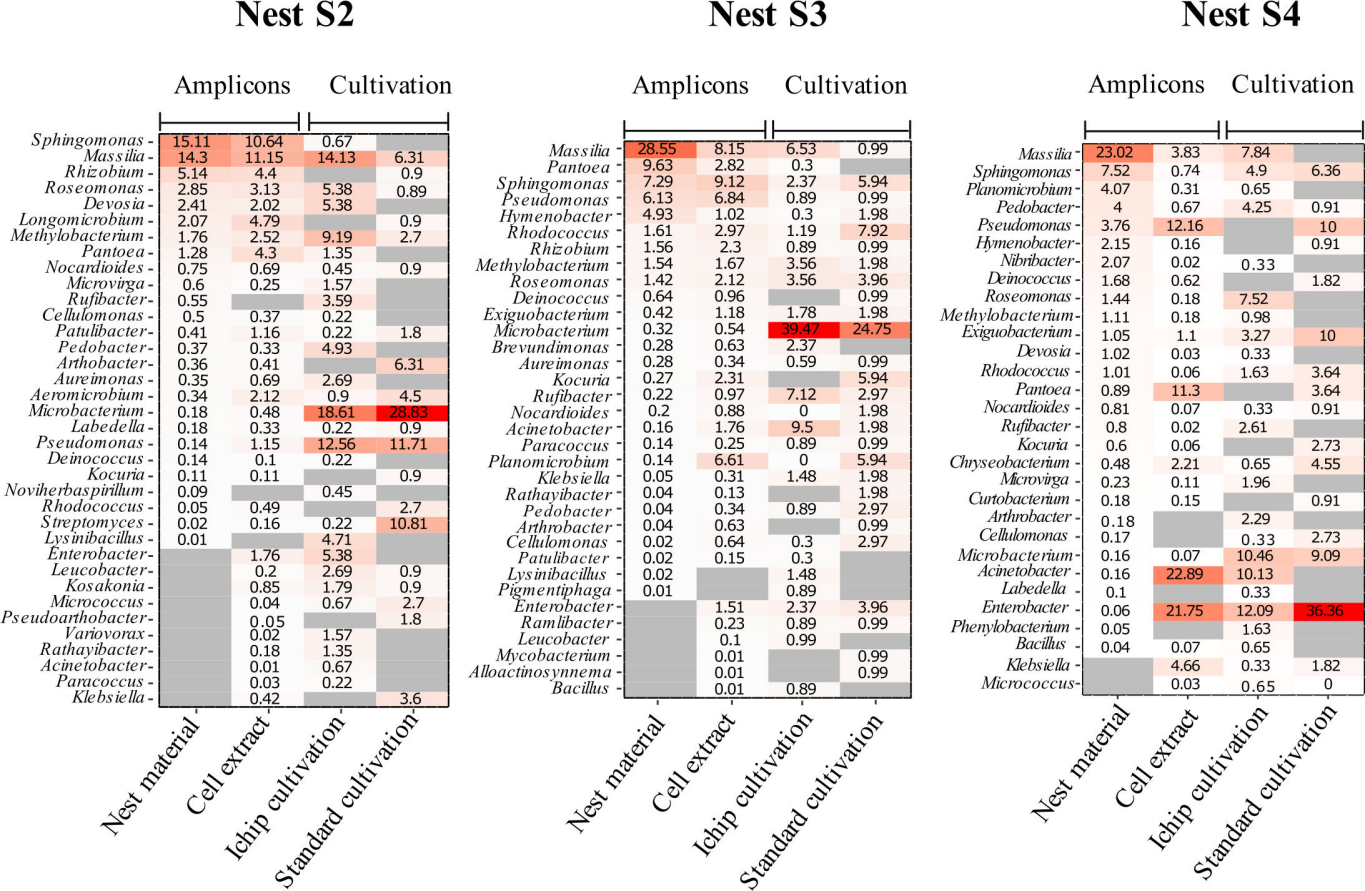
Relative abundance of genera shared between amplicon data and cultivation data. Amplicon data are shown separately for nest material and cell extracts and compared to iChip and standard cultivation. Heatmaps only include genera found in both amplicon data and cultivation data and are sorted according to abundance in the nest material amplicon data. Gray cells, a genus not detected. Note that relative abundance for amplicons represents 16S rRNA gene abundance, not cell abundance, while for cultivation it refers to the relative number of isolates.

Unlike in the original iChip study (12), where there was no overlap of bacterial taxa between iChip and standard cultivation, we found that one-third to two-thirds of all isolated genera could be retrieved with both methods (Fig. 3). However, our iChip data come from colonies grown on 1:10 NBA after only one round of *in situ* incubation, unlike for the original iChip studies that reported sequence data from iChip-grown microcolonies or “domesticated” isolates that underwent repeated cycles of *in situ* incubation (12, 15). Another difference to the original iChip study is that our standard isolation procedure utilized all three dilutions from the CFU plates and not only the one that was used for the iChip inoculation (Fig. 2). Therefore, our data indicate that inoculum size and downstream cultivation conditions still play an important role in defining the final cultivation outcome. Accordingly, more recent *in situ* cultivation studies (15, 28) have also reported overlap in genera and even species between standard and iChip isolation approaches.

Despite the overlap in the genera recovered by both the iChip and the standard cultivation in our study, each method resulted in the isolation of several unique genera (Fig. 3A); notably, the iChip recovered in total more than twice as many unique genera compared to standard cultivation (Fig. 3B). Our results thus indicate that the modified iChip resulted in an overall higher culturability, recovered a similar or higher fraction of the bacterial diversity from individual nests, and retrieved more unique genera compared to standard cultivation.

### Contamination risk and cultivation bias when using the iChip

When comparing the *in situ* bacterial communities of the nests with those found in the cell extracts and represented by the iChip and standard cultivation isolates (Fig. 3 and 4), several discrepancies are obvious.

First, for each nest, there was a small fraction of isolates (5%–14%) that had no resemblance to their original nest microbiome and/or cell extract. This could indicate that they were contaminants, introduced during the handling or incubation of the iChips. However, some of these “contaminant suspects,” like *Phenylobacterium* and *Lysinibacillus* (Fig. 4; Table S9), were found in the microbiome of one of the other nests, or, like *Gordonia* and *Providencia* (Table S4 and S9), have been earlier shown to be part of the spider nest microbiome (19). Their inconsistent detection may therefore rather reflect the nest microbiomes’ complexity and spatial heterogenicity within a nest (19). On the other hand, we did detect bacterial growth in the uninoculated control iChip, and some level of contamination seems inevitable considering all the steps involved in inoculation, incubation, and disassembly of the iChip (see Materials and Methods section and references 12,14 [29–32]). To our knowledge, this is the first study to include a negative control throughout the entire iChip *in situ* incubation period.

Second, already the cell extracts prepared from the original nest material had a slightly different community composition than the source material, with changes in both relative abundance and representation of both abundant and rare taxa (Fig. 4; Fig, S3). Obviously, both nest heterogeneity and cell-specific properties (e.g., determining how easily they can be dislodged or how well they survive sonication) play a role in skewing the isolation outcome even before the actual cultivation has started (33, 34).

Third, while the iChip performed only slightly better than standard cultivation for isolating the most abundant nest bacteria (Fig. 4; Fig. S3), including the nest core microbiome genera *Massilia* and *Sphingomonas* (19), it clearly outperformed the standard approach by isolating more rare genera with a relative abundance of less than 0.1% (Fig. 4; Table S9). This recovery of rare taxa is consistent with a recent study employing a modified iChip for the isolation of thermo-tolerant bacteria (15). Low-abundant genera uniquely growing in the iChips included *Lysinibacillus, Noviherbaspirillum, Phenylobacterium, Pigmentiphaga*, and *Variovorax* (Fig. 4; Table S4).

### VOCs as putative *in situ* substrates in the iChips

One intriguing question for the application of the modified iChip in dry conditions is as follows: what were the microcolonies in the iChip growing on besides the immediately available substrates from 1:10 NBA? The iChips had been inserted into the nest’s air space between dry nest material like spider silk, twigs, or leaves (Fig. S2), and while the constant water supply kept the agar plugs hydrated (Fig. 1), it did not allow transport of dissolved substrates into the growth chambers. Analyses of spider nests in the field, at three sites in Namibia, had detected 199 VOCs of which 53 could be identified they likely originated from bacteria, fungi, plants, and animals/animal carcasses in the nest (20). While some of the VOCs were shown to be antimicrobial (20, 25), others would be suitable as substrates for bacterial growth. In our laboratory nests, we found 19 VOCs of diverse chemical classes such as alcohols, organic acids, aldehydes, ketones, or aromatic compounds (Table S5). The much lower number of VOCs compared to the field samples is partially due to a different sampling and analysis pipeline (20), but likely also due to the absence of living plants and diverse prey animals in the laboratory setting. Yet, even this less diverse VOC mix contains a number of suitable microbial growth substrates, many of them likely produced by microbial degradation processes within the spider nest (35–37).

Some VOC, such as alcohols, formic acid, and acetic acid, are rather general substrates and might be used by many different bacterial taxa, while others, such as aromatic hydrocarbons or terpenes, require elaborate degradation pathways (38, 39) and their utilization is thus restricted to a few specialists. Interestingly, four of the unique iChip-derived genera (Table S4) contain known aromatic hydrocarbon degraders, that is, *Variovorax* (40), *Sphingopyxis* (41–43), *Phenylobacterium* (44, 45), and *Methylibium* (46–48); these genera were either rare or even absent in the molecular data set (Fig. 4), illustrating that specific *in situ* substrates may result in the isolation of rare specialists.

Other substrates that supported the growth of specialists in the iChips might be α-pinene, which certain *Nocardia* and *Bacillus* species can use as the sole carbon source (49, 50), or aliphatic aldehydes such as nonanal and decanal, also used by *Bacillus* (51). The latter VOCs were previously detected in spider nests in Namibia (25); they also have antimicrobial properties and might thus provide additional selective pressure during *in situ* cultivation.

### Field application and recovery of antimicrobial isolates

We deployed 20 modified iChips during a 2018 field trip into 10 nests of a *S. dumicola* population in Otavia, Namibia. Relative humidity within the nests ranged from 1 to 100% (mean, 51%), and temperature from 12.7**°**C to 55.7**°**C, with an average of 27.2**°**C (Table S6). Otavi was selected as the sampling site for antimicrobial isolates because nests from more humid regions, such as Otavi, have a higher risk of fungal infections than nests from dry regions (19). Thus, Otavi may offer a better opportunity to find antimicrobial isolates, as higher humidity may be selected for a stronger antimicrobial defense.

After 2 weeks of *in situ* incubation, we extracted approximately half of the agar plugs per iChip, with 82% of those agar plugs resulting in growth upon subculturing on standard growth medium. In total, we obtained 382 isolates from 49 genera, significantly extending the cultured diversity from *S. dumicola* nests (Table S7). Using a 16S rRNA identity cutoff of 98.7% (52), this collection includes (as a conservative estimate) at least 16 novel species and, most notably, 13 strains representing the three nest core microbiome genera *Massilia*, *Modestobacter*, and *Sphingomonas* (19) of which three strains showed antimicrobial activity (Table S8). Other *Massilia* isolates from spider nest microbiomes have previously been shown to produce VOCs with inhibitory effect against spider pathogens (25), as well as exhibiting antibacterial activity against Gram-negative human pathogens (53). *Brevundimonas* species were found to produce VOCs with nematocidal effect (54), suggesting that VOCs could be important contributors to the nest microbiome’s antimicrobial potential.

Overall, 19 of the isolates were active against at least one of the bacterial or fungal pathogens tested (Table S8). Taken together, these data demonstrate that the modified iChip can be used directly in the field to retrieve novel, ecologically relevant, and potentially biotechnologically interesting bacterial diversity.

### Conclusions and perspectives

The modified iChip presented in this study offers a method for *in situ* cultivation of microbes in arid environments. While neither a quantitative nor contamination-free tool, and requiring significant handling, the modified iChip allowed a higher recovery of the microbial diversity from social spider nests, bringing both dominant and rare microbiome members into the culture. Extending the cultured microbial diversity from *S. dumicola* nests is not only relevant for drug discovery but will help in understanding the role of the core microbiome in antimicrobial defense and ultimately the ecological success of the social spider *S. dumicola*.

For systems where miniaturization is not required (e.g., arid soils), a modified iChip design with larger agar plugs will reduce the risk of dehydration during disassembly and thus allow the picking and subculturing of individual microcolonies as a step toward a more quantitative and sterile procedure. In addition, the use of a more *in situ*-like medium (in our case e.g., chitin- or spider silk-based) inside iChips and during subculturing may further improve the cultivation of bacteria specific to a given environment.

## MATERIALS AND METHODS

### Design and construction of the modified iChip

The iChips were designed using the drawing program Autodesk Inventor v. 2018.3. The program sets out an output with codes that were fed into the computer-aided manufacturing software SolidCam 2018, which redirects the information to the CNC work center (Chevalier 1418VMC-40), producing several iChips at once. The exact dimensions of the top, central, and bottom iChip compartments are given in Fig. S1. All components of the modified iChip were constructed from polyoxymethylene (POM) (Nordisk Plast, Denmark). The central growth compartment was composed of a central through-hole plate covered by two sterile 0.03 µm polycarbonate filters (ø 25 mm) (GVS Life Science, USA), and two additional through-hole plates on each side. The through-hole plates were manually inspected, and edges formed by the drilling of the holes were removed using a sharp blade to create an even surface. The central growth compartment was sealed together using four bolts (L: 5 mm, Ø: 2 mm) and four nuts (W: 1 mm, Ø: 4 mm) (Fig. 1A, Panel 1). The central growth compartment was first attached to the top iChip plate using four larger bolts (L: 10 mm, Ø: 3 mm) and nuts (W: 1 mm, Ø: 6 mm) (Fig. 1A, panel 2), and finally to the bottom plate with hydration chamber using four additional nuts (W: 1 mm, Ø: 6 mm) (Fig. 1A, panel 3).

To ensure continuous water supply from the water reservoir (50 mL polypropylene centrifuge tube) to the hydration chamber of the iChip, a cotton thread (acting as a wick) was sucked through a 45 cm ISO-VERSINC tube (Saint-Gobain Performance plastics, France) using vacuum and inserted into the water reservoir at one end and into the hydration chamber at the other end. A hole was drilled in the lid of the 50 mL polypropylene centrifuge tube. using a 6 mm cork-borer to fit the ISO-VERSINC tube and a tight seal was made using silicon glue. Finally, a 27-gauge syringe needle (4 mm) was inserted into the lid of the 50 mL polypropylene centrifuge tube for pressure relief. The hydration chamber was mounted with two layers of microfiber cloth (cut to size) with the cotton wick (protruding from the ISO-VERSINIC tube) sandwiched between them (Fig. 1B, panel 2). The microfiber cloth served as an absorbing material, enabling the continuous flow of water through capillary forces from the hydration chamber during evaporation.

### Sampling of nest material for amplicon sequencing and cultivation

Nests were collected from Otavi, Namibia (19° 5′ 30.48″ S, 17° 13′ 43.752″ E) on 26 February 2019 and brought to Aarhus, Denmark. Nest material was sampled immediately after arrival (26 February 2019) using sterile forceps from three different areas of each nest. Nest material used for DNA extraction and sequencing was transferred to sterile 1.5 mL microcentrifuge tubes and stored at −20°C. Approximately 5 mL of nest material (on average 1.6 g) was collected for cell extraction in 50 mL sterile polypropylene centrifuge tubes and kept at 4°C.

### Cell extraction and enumeration

10 mL of 1× phosphate-buffered saline (PBS, pH 7.2) was added to the nest material in the 50 mL polypropylene centrifuge tube, and cells were dislodged by sonication (3 × 10 s at 10% intensity) (SONOPLUS, Bandelin, Germany) and vigorous vortexing (2 min), resulting in a homogeneous brownish slurry. The slurry was transferred to a 15 mL polypropylene centrifuge tube using a sterile 10 mL serological pipet, leaving larger particles (e.g., branches, leaves, and prey carcasses) behind. It was then left to settle for 2 min and pre-filtered through an 8 µm polycarbonate filter (ø 25 mm) (GE Water & Process Technologies, Netherlands) to remove fungal hyphae and spores. Cell extracts were then kept on ice during further processing to prevent growth before inoculation of iChips and agar plates.

Pre-filtered cell extracts were diluted 100×, and filters were prepared in triplicates for total cell counts: 1 mL diluted cell extract was mixed with 2 mL of sterile Milli-Q water and filtered through a 0.2 µm polycarbonate filter (ø 25 mm) (FRISENETTE, Denmark). Filters were washed with 70% ethanol, dried, transferred to microscopic slides, and stained with 1 µg mL^−1^ of 4′,6-diamidiono-2-phenylindole (DAPI) in CitiFluor (Electron Microscopy Science, USA) for 1.5 hours at 4°C. Cell counts were conducted in five random areas at 1,000× magnification using a Nikon Eclipse Ni-U fluorescence microscope.

### iChip loading and assembly

All iChip components were autoclaved, and inoculation and assembly were performed inside a laminar flow bench to minimize contamination. Based on the total cell counts, cell extracts were diluted to a final concentration of 10^3^ cells mL^−1^ in molten 1:10 NBA (0.08% wt/vol nutrient broth [Scharlau, Germany], 1.5% [wt/vol] Difco Bacto agar [BD, Germany]) in 50 mL polypropylene centrifuge tube. The inner growth plate of the iChip was dipped into the liquid agar-cell mix and transferred to a sterile 50 mL polypropylene centrifuge tube to allow the agar to solidify inside each through-hole. Using a sterile scalpel, excess agar was scraped off, and the inner growth plate was sandwiched between polycarbonate filters and the two additional through-hole plates as shown in Fig. 1. Then the micro-fiber cloth positioned in the bottom plate was primed with sterile Milli-Q water to initiate the capillary force, and the iChip assembly was concluded by attaching the outer and central growth compartments to the bottom plate (Fig. 1).

### Cultivation and isolation

The cultivation experiment was started on 29 March 2019, with nest S2, followed by nest S3 and S4 on 5 April 2019 by inserting each loaded iChip into the nest from which its cell extract had been prepared (Fig. S2). At the same time, 100 µL of the cell extracts were plated onto 1:10 NBA plates, in three different dilutions and in triplicate, except for nest S2, where one iChip was used as negative control and left uninoculated (Fig. 2). Both nests (with iChips) and 1:10 NBA plates were kept for 2 weeks in a 13:11 light cycle with a fluctuating temperature from 20° (night) to 29°C (day) to resemble the natural conditions. The 24 hour temperature cycle was as follows: A gradual increase from 20°C to 24°C (1 hour duration), followed by another gradual increase from 24°C to 29°C (1 hour duration), 10 hours at 29°C, a gradual decrease from 29°C to 24°C (1 hour duration), and a second gradual decrease from 24°C to 20°C (1 hour duration), and finally 20°C for 10 hours. Every second day, the iChip water reservoirs were checked and filled with sterile Milli-Q water if necessary.

After 2 weeks, iChips were washed with particle-free DNA-grade water, air-dried, and disassembled. Agar plugs were plugged out from through-holes using an unfolded sterile paper clip and transferred directly to microcentrifuge tubes containing 150 µL 1× PBS. The agar-plug extraction was carried out in a 4°C room to slow down the desiccation of the agar plugs. Agar plugs were disrupted and homogenized by vortexing (20 s) and by pipetting up and down; then, 100 µL of the homogenate was spread onto 1:10 NBA plates, which was incubated at 30°C until colonies appeared.

Colonies were selected based on differences in colony morphology, and subcultured onto 1:10 NBA plates at 30°C, first for 2 weeks, and then at 7-day intervals until pure cultures were obtained. Pure cultures were categorized into morphotypes based on colony shape, coloration, texture, elevation, and opacity. At least one isolate representing a unique morphotype was tentatively identified by 16S rRNA gene sequencing. The number of isolates in each morphotype category was counted and noted down. After identification, isolates were grouped to new morphotypes, as several morphotypes were assigned to the same phylotype. The isolate count was normalized by fractionation and expressed as percentages. Isolates were preserved in 1:10 NBA supplemented with 50% glycerol and stored at −80°C.

### Identification of isolates

Templates for colony PCR were prepared from single colonies dissolved in 20 µL of sterile RNase/DNase-free water (Qiagen, Germany). The PCR master mixture contained 10.5 µL dH_2_O, 12.5 µL Taq DNA Polymerase Master Mix RED (Ampliqon, Denmark), 0.5 µL of BSA (20 mg/mL), and 0.5 µL of primers 6F (55) and Bac1075R (56) (10 pmol/µL), and finally 0.5 µL template or nuclease-free water as a negative control. Amplification was achieved by the following thermocycling scheme: Initial denaturation and cell lysis at 95°C for 15 min, 35 PCR cycles of 94°C for 45 s, 57°C for 45 s, and 72°C for 1 min, and a final elongation step of 10 min at 72°C. The DNeasy Blood & Tissue kit “Gram-positive bacteria protocol” (Qiagen, Germany) was used for the extraction of DNA from isolates, where DNA amplification was not achieved using the colony PCR procedure as described above. PCR products were sequenced by Macrogen Europe B.V., and the obtained partial 16S rRNA gene sequences were trimmed in Geneious v.11.0.4 (https://www.geneious.com) using the “Trim ends” function with default settings. Trimmed sequences were blasted to the NCBI 16S ribosomal RNA database using the built-in BLAST function in Geneious v. 11.0.

### DNA extraction, 16S rRNA gene amplicon sequencing, and analysis

DNA was extracted from nest material and cell extracts using the DNeasy Blood & Tissue Kit (Qiagen) according to the manufacturer’s protocol with the following modifications: samples were first crushed in liquid nitrogen with a pellet pestle (Sigma-Aldrich) and further homogenized with the pestle after the addition of the ATL buffer. DNA extracts from triplicate nest material were pooled, yielding one DNA extract from each nest containing DNA from all three subsamples.

16S rRNA gene amplicon libraries were prepared according to Illuminas’s 16S Metagenomic Sequencing Library Preparation guide using the primers 341F (5′-CCTACGGGBGGCWGCAG-3′) and 805R (5′-GACTACHVGGGTATCTAATCC-3′) to amplify the variable V3-V4 region (57). PCR was performed with the following program: Initial denaturation at 95°C for 3 min followed by eight cycles at 95°C, 55°C, 72°C of 30 s each, and a final extension at 72°C for 3 min. All PCRs were performed on a Veriti 96-Well Thermal Cycler (Applied Biosystems) and after each PCR products were purified using AMPure XP magnetic beads. DNA concentrations were measured using the Quant-iT dsDNA BR assay kit on a FLUOstar Omega fluorometric microplate reader (BMG LABTECH, Germany). DNA was diluted to a final concentration of 3 ng mL^−1^. DNA extracts were pooled and sequenced on an Illumina MiSeq desktop sequencer.

All data processing was done in RStudio v. 1.0.153 using customized R scripts provided in the supplementary information. Primers were trimmed from raw reads using the package Cutadapt v.1.18 (58). Filtering, de-replication, merging of paired-end reads, removal of chimeras, and classification were done using the R package “dada2” (59) following filter settings: maxEE = 2.2, truncQ = 2, and truncLen = 230, and Silva SSU reference database nr. 132 for classification. ASV-, taxonomy-, and sample tables were extracted using the R package “phyloseq” (60). Amplicon sequence variants (ASVs) were filtered to exclude ASVs <400 bp and ASVs not classified as bacteria. Amplicon reads were normalized by fractionation of all reads per sample. The R packages “dplyr” v. 0.8.5 (61) and “ggplot2” v. 3.3.0 (62) were used for ASV summation to genus-level and heat-map visualization.

### Nest atmosphere sampling

Nest atmospheres were sampled in the laboratory on 27 March 2019 with adsorbent tubes (containing Tenax TA, Carbopack B, and Carboxen 1003, Sigma Aldrich, USA), which prior to use were conditioned in a tube conditioner (TC-20, Markes International) at 300°C for 30 min. Afterward, tubes were stored in closed glass containers at −20°C until use. Air sampling was conducted with an SKC Pocket Pump TOUCH (SKC Inc., USA) that had been calibrated prior to sampling and was set to a flow of 50 [+2] mL min^−1^ giving a flow of 50.34 mL min^−1^. The pump was calibrated again after use resulting in a flow of 50.2 mL min^−1^. The mean of the two calibration flows was used for the subsequent analyses. The pump collected the nest atmosphere for 10 min, resulting in a total sampling volume of 503.4 cm^3^. The approximate volume of each nest was estimated to control for oversampling. The approximate volume of nest S2 was 1017 cm^3^, 727 cm^3^ for nest S3, and 499 cm^3^ for nest S4, respectively. In addition, a background sample of 503.3 cm^3^ of laboratory atmosphere was collected. The temperature in the laboratory on the day of sampling was measured continuously and ranged over the sampling period from 23.7 to 25.3°C.

### Gas chromatography coupled to mass spectroscopy

For loading of samples onto the gas chromatography (GC) column, the adsorbent tubes (with sample) were thermally desorbed at 300°C for 10 min. The start temperature was 20°C and the temperature increased with a rate of 120°C min^−1^. The desorption flow was 50 mL min^−1^ and the split ratio for the thermal desorption was 0.1:1. Desorbed analytes were collected by cold trapping with Cooled Injection System (*CIS*) (Gerstel, Germany) and trapped on a quartz wool liner using a liquid N_2_ at −100°C. By heating the *CIS* to 275°C at a rate of 12°C s^−1^, the trapped analytes were released onto the GC column. The hold time was 10 min and the split ratio for the *CIS* was 9:1. Separation of analytes was obtained on a 28.6 m (0.25 mm × 0.25 mm) RTX-200MS column with a Crossbond trifluoropropylmethyl polysiloxane stationary phase (Restek, France). Helium was used as carrier gas with a 1 mL min^−1^ flow. The column oven program started at 35°C with a hold time of 2 min. Column temperature increased by 10°C min^−1^ to 300°C. The hold time was 3 min, which gave a total run time of 31.5 min. The MS had a solvent delay of 1.5 min and the ion source was kept at 250°C. The temperature of the quadrupole was kept at 150°C. The MS scanned from 30 to 500 m/z.

### Data processing of GC-MS data

GC-MS data were processed with Unknowns Analysis in Mass Hunter version B 8.00 software (Agilent, USA). Peaks were annotated from raw data by the deconvolution method and identified *via* library search in the NIST-11 mass spectral database 2.0 d (National Institute of Standards and Technology, USA). The compounds were only included in the analysis if the match factor was above 90% in the Unknown Analysis.

### Field application of the modified iChip

A total of 20 modified iChips (two iChips per nest) were deployed during a field campaign to Otavi, Namibia, in January 2018. On average, 0.64 g (5 mL) of nest material was collected from each nest in the field using ethanol-washed tweezers and scissors, which were cleaned between each sampling event. The collected nest material was stored in sterile 15 mL polypropylene centrifuge tubes and transported back to the field laboratory on the same day for subsequent cell extraction, following the procedure described in the previous method section (Cell extraction and enumeration) with minor modifications. Notably, the cell extracts were prepared from a slurry created from shaking the nest material up and down in 10 mL sterile PBS combined with 3 × 20 s of vigorous vortexing. Half of the extracts were diluted to a final concentration of 10^3^ cells mL^−1^ in molten 1% Difco Bacto agar (BD, Germany) while the other half was diluted similarly in molten 1% Difco Bacto agar (BD, Germany) supplemented with 0.5% yeast extract (Sigma-Aldrich). Inoculated iChips were reinserted in their source nests while the water reservoirs connected to the iChips were placed inside nylon socks and secured to nearby branches situated slightly above the nests (Fig. S4). Ten iButton temperature and humidity loggers (Maxim Integrated, CA, USA) were used to monitor the ambient temperature and humidity both inside and outside of five nests during the 2-week incubation period (Table S6). The water reservoirs were inspected for water loss every second day, and if necessary refiled with pre-boiled tap water.

After 2 weeks of *in situ* cultivation, each iChip-containing nest was cut down from the branch and placed inside a plastic box (L: 195 mm × B: 195 mm × H: 110 mm) together with the two water reservoirs. The boxes with the nests and the iChips were transported back to Aarhus University, Denmark. Upon arrival, the iChips were removed from the nests and placed inside a sealed box containing dampened paper at the bottom to prevent the agar from desiccating. The box was stored at 4°C for ~5 days before proceeding with disassembly and agar-plug extraction, which was carried out as described in the previous method section (Cultivation and isolation), with the exception that the agar plugs were plated out onto standard (undiluted) NBA plates (8 g L^−1^ NB) and R2A agar plates in addition (Neogen, UK). Subculturing, isolation, and identification of the isolates were conducted following the same criteria as described for the laboratory experiment.

### High-throughput antimicrobial activity screening field iChip isolates

The antimicrobial activity was determined by three different assays: (i) the antagonistic assay (A), (ii) soft agar-overlay assay (O), and (iii) using crude extracts (CE) obtained from cell-free supernatants extracted three times with 1:1 with ethyl acetate, concentrated by rotary evaporation, and dissolved with 1 mL 100% MeOH. The pathogenic test strains included *E. coli* ATCC 11229. *Staphylococcus aureus* ATCC 6538, *Pseudomonas aeruginosa* ATCC 27853, *Enterococcus facium* ATCC 19434, *Acinetobacter baumannii* ATCC 19606, *Klebsiella pneumoniae* ATCC 13883, and *Candida albicans* CCUG 59850.

Prior to antimicrobial screening, iChip isolates were inoculated into 96-well plates (SARSTEDT, Germany) containing 150 µL NB medium in each well. The isolates were incubated at 30°C under agitation (100 rpm) for 24 hours before being point-inoculated onto 1% (wt/vol) NB agar plates (150 mm × 15 mm) using a microplate pin replicator to test for differences in growth properties. The isolates were categorized into different growth types (Spreaders, fast- and slow-growing isolates) and re-inoculated onto new 96-well plates according to growth characteristics before being re-incubated for another 24 hours under the same culture conditions as previously. Stocks were prepared by adding 150 µL 99% glycerol (Sigma-Aldrich, USA) to the 96-well plates and stored at −80°C.

The test plates used for the antagonistic assay were prepared according to the Kirkby-Bauer Disk Diffusion Susceptibility Test protocol (63) with some modifications to accommodate bacterial testing instead of antibiotics. Colonies of pathogenic test strains were suspended in 5 mL sterile 0.9% saline water with the absorbance adjusted to OD_600_ = 0.010 for the fast-growing test strains (*E. coli, S. aureus, P. aeruginosa*, and *A. baumannii*) or OD_600_ = 0.10 for more slow-growing test strains (*C. albicans*, *E. facium*, and *K. pneumonia*). The pathogens were spread onto (150 mm × 15 mm) Mueller Hinton (Sigma-Aldrich, USA) agar (MHA) and dried for 15 min in a Laminar Flow Bench before being point-inoculated with iChip isolates using the plate replicator. The plate replicator was washed with 70% ethanol and flame-sterilized between every inoculation onto the different test plates.

For the soft agar-overlay assay, the iChip isolates were point-inoculated onto 1:10 NBA and incubated for 3 days before the overlay of 10 mL 0.5% (wt/vol) molten MHA mixed with the test pathogenic strains diluted to the same cell concentration as used for the antagonistic assay. The test plates for antimicrobial screening of crude extracts were made by mixing 1 µL of overnight cultures of the cultures per mL of melted MHA. Holes were punctured into the solidified agar, using the back end of a P1000 pipette tip and 50 µL of crude extracts from specific iChip isolates (Table S8) was added to the holes. 50 µL of crude extracts prepared from bacterial-free growth medium and 50 µL 100% MeOH were used as controls. The inoculated plates were incubated at 30°C and examined for inhibition zones every 24 hours.

## Data Availability

Amplicon sequences from nest material and cell extracts were submitted to NCBI Sequence Read Archive (SRA) with the BioProject accession number PRJNA685850. Partial 16S rRNA gene sequences were submitted to the NCBI GenBank with the accession numbers MT803595-MT804295.

## SUPPLEMENTAL MATERIAL

Supplemental material aem.01325-24-s0001.pdf Tables S1 to S8, Figures S1 to S4, and R code used for data analysis.

Table S9 aem.01325-24-s0002.xlsx Identification and relative abundance of all ASVs present in nest material and cell extracts.

ASM does not own the copyrights to Supplemental Material that may be linked to, or accessed through, an article. The authors have granted ASM a non-exclusive, world-wide license to publish the Supplemental Material files. Please contact the corresponding author directly for reuse.
