# Supplementary material for: A modified iChip for in situ cultivation of bacteria in arid environments
Source: Appl Environ Microbiol. 2025 Jan 8;91(2):e01325-24. doi: 10.1128/aem.01325-24 (PMC11837541; doi:10.1128/aem.01325-24)
Supplement: Supplemental material — Tables S1 to S8, Figures S1 to S4, and R code used for data analysis. [file aem.01325-24-s0001.pdf]

Supplementary Information for:

A modified iChip for *in situ* cultivation of bacteria in arid environments

Seven Nazipi Bushi[1*], Marie B. Lund[1], Tobias Sandfeld[1], Sanne Sadolin Nørskov[3], Simon Fruergaard[3], Marianne Glasius[3], Trine Bilde[2], and Andreas Schramm[1]

[1]*Department of Biology, Section for Microbiology, Aarhus University, DK*
[2]*Department of Biology, Section for Genetics, Ecology and Evolution, Aarhus University, DK*
[3]*Department of Chemistry, Aarhus University, DK*

*Corresponding author: Seven Nazipi Bushi; E-mail: Seven90@bio.au.dk

Contents:

Table S1. Culturability of bacterial cells recovered from standard cultivation.

Table S2. Number of isolates retrieved by iChip and standard cultivation per nest.

Table S3. Bacterial diversity per nest retrieved by amplicon sequencing and cultivation.

Table S4. Unique genera and genera shared between cultivation methods.

Table S5. Volatile organic compounds (VOCs) in the laboratory iChip nest atmosphere.

Table S6. GPS coordinates, temperate, and relative humidity (% RH) for nests from iChip field application

Table S7. Best BLAST hit of isolates recovered from iChips incubated in *S. dumicola* spider nests in the field (Otavi, Namibia).

Table S8. Activity of antimicrobial iChip isolates against *ESKAPE* pathogens and *Candida albicans*

Table S9. (extra file) Excelfile with the taxonomic identification and abundance of ASVs recovered from nest material and cell extracts.

Fig. S1. Schematic drawings of the three iChip components created with Autodesk Inventor.

Fig. S2. Photographs of the laboratory setup during in situ incubation with the modified iChip.

Fig. S3. Relative abundance of the 25 most abundant nest microbiome members and their representation in cultivation data.

Fig. S4. In situ cultivation with the modified iChip during field campaign in Otavi, Namibia.

Supplemetary Information: R codes used for data analysis

Table S1. Culturability of bacterial cells recovered from standard cultivation.

Nest	Mean TCC ± SD (Cells mL^{-1})	Mean CFU[a] ± SD (Cells mL^{-1})	Culturability (± SD)
**S2**	4.53×10^{8} (± 4.14×10^{7})	6.13×10^{6} (± 3.40×10^{5})	1.35% (± 0.08%)
**S3**	4.48×10^{7} (± 6.94×10^{6})	1.01×10^{6} (± 3.67×105)	2.26% (± 0.82%)
**S4**	2.69×10^{7}(± 2.75×10^{6})	9.39×10^{5} (± 3.46×105)	3.49% (± 1.39%)
**Mean**	1.75×10^{8} ± 2.41×10^{8}	2.69×10^{6} ± 2.16×10^{6}	2.37% (± 1.37%)

[a] Mean CFU was calculated from plates containing >100 CFU.

Table S2. Number of isolates per nest retrieved by iChip and standard cultivation.

Nest	Isolates	Morphotypes[a]	Phylotypes[b]	Genera
**S2**				
iChip	450[a]	60	59	35
Standard	112	39	41	25
**S3**				
iChip	341[a]	64	63	33
Standard	102	51	51	33
**S4**				
iChip	316[a]	56	57	33
Standard	111	32	32	19

[a] Number of morphotypes after re-categorization based on 16S rRNA identities.
[b] Number of unique 16S rRNA sequences.

Table S3. Bacterial diversity per nest retrieved by amplicon sequencing and cultivation.

Nest	Nest material (ASV/genera)	Cell extracts (ASV/genera)	iChip (phylotypes/genera)	Standard cultivation (phylotypes/genera)
**S2**	540/139	377/129	59/35	41/25
**S3**	462/135	606/166	63/33	51/33
**S4**	488/152	183/91	57/33	32/19
**TOTAL[a]**	1118/230	892/209	158/62	112/48

[a] Number of unique ASV, phylotypes, and genera summed over all samples.

Table S4. Unique genera and genera shared between cultivation methods.

	iChip cultivation	*Standard cultivation*	*Shared*
**# Genera**	22	9	37
**Genera**	*Agrococcus*<br>*Arenivirga*<br>*Bacillus*<br>*Brevundimonas*<br>*Bosea*<br>*Gordonia*<br>*Devosia*<br>*Dietza*<br>*Lysinibacillus*<br>*Methylibium*<br>*Microvirga*<br>*Neorhizobium*<br>*Nibribacter*<br>*Nocardia*<br>*Noviherbaspirillum*<br>*Phenylobacterum*<br>*Pigmentiphaga*<br>*Pontibacter*<br>*Provedencia*<br>*Sphingopyxis*<br>*Staphylocoocus*<br>*Variovorax*	*Alloactinosynnema*<br>*Curtobacterium*<br>*Cellulosemicrobium*<br>*Cronobacter*<br>*Curvibacter*<br>*Kocuria*<br>*Longimicrobium*<br>*Mycobacterium*<br>*Pseudoarthobacter*	*Acidovorax*<br>*Acinetobacter*<br>*Aeromicrobium*<br>*Arthrobacter*<br>*Aureimonas*<br>*Cellulomonas,*<br>*Chryseobacterium*<br>*Deinococcus*<br>*Dermacoccus*<br>*Enterobacter*<br>*Exiguobacterium*<br>*Hymenobacter*<br>*Klebsiella*<br>*Kosakonia*<br>*Labedella*<br>*Leucobacter*<br>*Massilia*<br>*Methylobacterium*<br>*Methylorubrum*<br>*Microbacterium*<br>*Micrococcus*<br>*Mixta*<br>*Nocardioides*<br>*Pantoea*<br>*Paracoccus*<br>*Patulibacter*<br>*Pedobacter*<br>*Planomicrobium*<br>*Pseudomonas*<br>*Ramlibacter*<br>*Rathayibacter*<br>*Rhizobium*<br>*Rhodococcus*<br>*Roseomonas*<br>*Rufibacter*<br>*Sphingomonas*<br>*Streptomyces*

Table S5. Volatile organic compounds (VOCs) in the laboratory iChip nest atmosphere. Compounds detected qualitatively are annotated with an x and a Match factor, while compounds identified semi-quantitatively with PARADISe have concentrations assigned. Compounds identified both qualitatively and semi-quantitatively have a match factor and a concentration assigned. *Background* refers to compounds detected in the laboratory atmosphere.

Compound	*Class*	*Molecular formula*	*RT[a] (min)*	*Match[b] factor (%)*	*Nest S2*	*Nest S3*	*Nest S4*	*Background*
					Concentration ($\mu g\ m^{-3}$)			
**Methanol**	Alcohol	CH_3OH	2.05	95.12	185.6	37.5	63.3	*x*
**Ethanol**	Alcohol	C_2H_5OH	2.17	95.90	394.9	278.3	193.8	*x*
**Acetonitrile**	Nitrile	CH_3CN	2.71	98.07	*x*	*x*	*x*	*N.D.*[c]
**Benzene**	Benzene	C_6H_6	2.78	94.85	0.1	0.3	2.8	*x*
**Formic acid**	Carboxylic acid	$CHOOH$	2.83	98.30	*N.D.*	x	*N.D.*	*N.D.*
**Butanol**	Alcohol	C_4H_9OH	3.08	-	3.2	3.8	2.7	*N.D.*
**Acetic acid**	Carboxylic acid	CH_3COOH	3.18	98.10	25.6	68.9	21.6	*x*
**Toluene**	Toluene	$C_6H_5CH_3$	3.71	94.73	9.3	12.1	13.9	*N.D.*
**Hydroxy acetone**	Propanone	$C_3H_6O_2$	4.60	96.45	*N.D.*	28.3	*N.D.*	*N.D.*
***α*-pinene**	Terpene	$C_{10}H_{16}$	5.24	-	0.4	0.6	0.3	*N.D.*
***o*-cymene**	Aromatic hydrocarbon	$C_{10}H_{14}$	6.99	-	0.3	2.1	0.2	*N.D.*
**Benzaldehyde**	Aldehyde	C_6H_5CHO	8.07	-	2.2	2.2	1.1	*N.D.*
**Nonanal**	Aldehyde	$C_9H_{18}O$	9.77	-	0.7	*N.D.*	1.6	*N.D.*
**2-Furanone**	Butenolide	$C_4H_4O_2$	9.68	-	*N.D.*	6.5	*N.D.*	*N.D.*
**Acetophenone**	Ketone	$C_6H_5COCH_3$	9.88	-	*N.D.*	*N.D.*	0.2	*N.D.*
**Benzoic acid**	Carboxylic acid	C_6H_5COOH	10.06	-	*N.D.*	4.2	1.3	*N.D.*
**Decanal**	Aldehyde	$C_{10}H_{20}O$	11.09	-	0.6	*N.D.*	1.4	*N.D.*
**N-hexa-decanoic acid**	Fatty acid	$C_{16}H_{32}O_2$	18.47	95.15	*N.D.*	209.8	*N.D.*	*N.D.*
**Octadecanoic acid**	Fatty acid	$C_{18}H_{36}O_2$	20.23	-	*N.D.*	13.9	0.8	*N.D.*

[a] RT, retention time in minutes
[b] Match factor, refers to the similarity (in %) of the measured mass spectrum with the mass spectrum of the NIST11 MSsearch library (NIST: National Institute of Standards and Technology, USA).
[c] N.D., compound not detected.

Table S6. GPS coordinates, and inside/outside temperate (°C) and relative humidity (% RH) for nests from iChip field application.

**Nest**	**location**		**iChip**	**% RH**						**Temperature (°C)**					
	**GPS coordinates**			**inside**			**outside**			**inside**			**outside**		
	**latitude**	**longitude**		Min	Avg	Max	Min	Avg	Max	Min	Avg	Max	Min	Avg	Max
**H129S**	-19.47726	17,19410	X												
**H130S**	-19,47741	17,19422	X												
**H131S**	-19,47712	17,19424	X												
**H137S**	-19,47829	17,19487	X	4.5	55.5	100	11.2	55.6	100	12.7	26.3	45.5	16.0	27.3	43.4
**H139S**	-19,47875	17,19512	X	1.2	43.6	100	8.6	55.3	100	13.9	29.8	55.7	17.6	24.9	45.9
**H141S**	-19,47908	17,19507	X												
**H142S**	-19,47844	17,19364		2.1	61.2	100	14.1	55.8	100	13.1	26.5	52.3	20.4	25.7	45.0
**H143S**	-19,47900	17,19491	X												
**H146S**	-19,47895	17,19339	X												
**H147S**	-19,47921	17.4102	X												
**H148S**	-19,47950	17,19329	X												
**H149S**	-19,47815	17.4102		7.4	54.1	99.8	27.6	45.9	98.9	13.4	24.9	41.8	22.2	22.9	39.9
**H153**	-19,47998	17,19456		1.05	42.6	100	1.06	49.4	100	13.8	28.6	52.2	13.8	24.1	38.6
**Mean**				**3.3**	**51.4**	**99.9**	**12.5**	**52.4**	**99.8**	**13.4**	**27.2**	**49.5**	**18**	**25**	**42.6**

Table S7. Best BLAST hit of isolates recovered from iChips incubated in *S. dumicola* spider nests in the field (Otavi, Namibia).

**Isolates**	**Best BLAST hit***	**% Identity**
IC-348, IC-313, IC-24, IC-210	*Acidovorax delafieldii* strain 133	99.4
IC-39, IC-40, IC-64, IC-74, IC-85	*Acinetobacter dijkshoornia*e strain JVAP01	99.6
IC-106, IC-28	*Acinetobacter indicus* strain A648	99.6
IC-121, IC-127, IC-84, IC-132, IC-14	*Acinetobacter lwoffii* strain DSM 2403	99.4
IC-186, IC-23, IC-351, IC-355, IC-82, IC-89	*Acinetobacter radioresistens* strain NBRC 102413	98.8
IC-7, IC-76	*Acinetobacter schindleri* strain LUH5832	100
IC-328	*Acinetobacter vivianii* strain NIPH 2168	99.7
IC-234, IC-65, IC-70, IC-71, IC-72, IC-73	*Agrobacterium fabrum* strain C58	100
IC-21, IC-83	*Agrobacterium larrymoorei* strain AF3.10	98.3
IC-264	*Agrococcus jenensis* strain DSM 9580	99.3
IC-374, IC-9	*Cryobacterium ruanni* strain Sr36	99.3
IC-68	*Aquincola tertiaricarbonis* strain L10	98.8
IC-214, IC-226	*Arthrobacter crystallopoietes* strain DSM 20117	99.5
IC_206	*Aurantimonas endophytica* strain EGI 6500337	99.8
IC-266, IC-212	*Aurantimonas frigidaquae* strain CW5	97.6
IC-131	*Bacillus circulans* strain NBRC 13626	95.1
IC-81	*Bacillus foraminis* strain CV53	99.1
IC-185	*Bacillus halosaccharovorans* strain E33	99.0
IC-199, IC- 209	*Bacillus nealsonii* strain DSM 15077	99.7
IC-120	*Bacillus tropicus* strain MCCC 1A01406	99.8
IC-356	*Bacillus wiedmannii* strain FSL W8-0169	96.6
IC-122, IC-154	*Bordetella bronchialis* strain AU3182	99.2
IC-196, IC-20, IC-12	*Brevibacterium frigoritolerans* strain DSM 8801	99.9
IC-107, IC-11	*Brevundimonas intermedia* strain ATCC 15262	99.9

IC-205	*Brevundimonas lenta* strain DS-18	94.3
IC-1, IC-2, IC-17, IC-33, IC-37, IC-43, IC-44, IC-5	*Brevundimonas terrae* strain KSL-145	98.4
IC-54, IC-62, IC-151, IC-17, IC-171, IC-284, IC-103, IC-240, IC-248, IC-257, IC-94, IC_96, IC_98	*Brevundimonas vesicularis* strain NBRC 12165	98.4
IC-301	*Brevundimonas staleyi* strain FWC43	99.5
IC-376	*Cellulosimicrobium cellulans* strain DSM 43879	99.9
IC-363	*Cellulosimicrobium marinum* strain RS-7-4	98.9
IC-123	*Chryseomicrobium imtechense* strain MW 10	99.7
IC-146	*Chryseomicrobium palamuruense* strain PU1	99.2
IC-15, IC-3, IC-36	*Comamonas sediminis* strain S3	100
IC-13	*Cupriavidus basilensis* strain DSM 11853	97.9
IC-32, IC-79	*Cupriavidus respiraculi* strain AU3313	99.2
IC-217	*Delftia lacustris* strain 332	99.8
IC-268	*Devosia riboflavina* strain NBRC 13584	99.8
IC-317, IC-319, IC-66, IC-67	*Devosia* submarina strain Sl74	99.5
IC-276	*Exiguobacterium acetylicum* strain DSM 20416	99.3
IC-175, IC-179, IC-182, IC-219	*Exiguobacterium aurantiacum* strain NBRC 14763	99.5
IC-97	*Herbaspirillum seropedicae* strain NBRC 102524	99.6
IC-331	*Kocuria rhizophila* strain TA68	99.1
IC-190, IC-378	*Kocuria rosea* strain DSM 20447	99.5
IC-159, IC-321	*Kocuria sediminis* strain FCS-11	100
IC-271	*Labedella gwakijensis* strain KSW2-17	99.6
IC-346, IC-340	*Leclercia adecarboxylata* strain NRBC 102595	99.7
IC-108, IC-150, IC-41, IC-50	*Leucobacter chromiiresistens* strain JG 31	99.2
IC-285	*Massilia aurea* strain AP13	98.3
IC-270	*Massilia niastensis* strain 5516S-1	98.8
IC-4, IC-56	*Massilia suwonensis* strain 5414S-25	99.2
IC-45, IC-77	*Massilia timonae* strain UR/MT95	99.3
IC-342, IC-19, IC-192, IC-215, IC-227	*Massilia varians* strain CCUG 35299	99.5
IC-327	*Methylorubrum aminovorans* strain JCM 8240	100
IC-289	*Methylobacterium goesingense* strain iEII3	98.7
IC-293	*Methylobacterium terrae* strain 17Sr-1-28	99.4
IC-291, IC-292	*Methylobacterium indicum* strain SE2.11	98.7
IC-233	*Microbacterium aurantiacum* strain C820	99.9
IC-241	*Microbacterium chocolatum* strain DSM 12507	99.8
IC-224	*Microbacterium desertii* strain SYSU D8014	99.4
IC-118, IC-280	*Microbacterium esteraromaticum* strain DSM 8609	98.9
IC-279, IC-329	*Microbacterium flavum* strain YM18-098	99.2
IC-144-145, IC-195, IC-202, IC-230, IC-24, IC-31, IC-362, IC-87, IC-95	*Microbacterium hydrocarbonoxydans* strain BNP48	100
IC-16, IC-42, IC-51, IC-29, IC-88	*Microbacterium paraoxydans* strain CF36	99.5
IC-191	*Microbacterium proteolyticum*	99.4
IC-144	*Microbacterium saperdae* strain IFO 15038	99.6
IC-119, IC-46	*Microbacterium schleiferi* strain DSM 20489	100
IC-12, IC-24, IC-8, IC-173	*Micrococcus flavus* strain LW4	99.1
IC-142	*Micrococcus terreus* strain V3M1	99.6
IC-130, IC-176, IC-47	*Micrococcus yunnanensis* strain YIM 65004	99.7
IC-204	*Micromonospora noduli* strain GUI43	98.9

IC-344	*Microvirga subterranea* strain Fail4	98.6
IC-249	*Modestobacter versicolor* strain CP153-2	99.5
IC-60	*Myxococcus fulvus* strain ATCC 25199	99.0
IC-101, IC-354, IC-369, IC-52, IC-90, IC-91, IC-92-93	*Paenibacillus chitinolyticus* strain NBRC 15660	99.5
IC-115, IC-312	*Paracoccus acridae* strain SCU-M53	100
IC-260	*Paracoccus acridae* strain SCU-M53	98.4
IC-253	*Paracoccus chinensis* strain NBRC 104937	100
IC-299	*Paracoccus marcusii* strain MH1	100
IC-168, IC-213	*Paracoccus marinus* strain NBRC 100637	99.8
IC-129, IC-152, IC-155, IC-315	*Paracoccus sanguinis* strain 05503	99.5
IC-223	*Paracoccus speluncae* strain 0911TES13M5	100
IC-238, IC-360	*Planococcus massiliensis* strain ES2	99.8
IC-112, IC-357	*Planococcus ruber* strain CW1	99.9
IC-113, IC-160	*Planomicrobium chinense* strain DX3-12	99.9
IC-105, IC-109, IC-114	*Planomicrobium okeanokoites* strain NBRC 12536	99.3
IC-133, IC-254, IC-278, IC-322	*Planomicrobium soli* strain XN13	99.7
IC-172, IC-38	*Prolinoborus fasciculus* strain CIP 103579 1	99.2
IC-78	*Pseudomonas chengduensis* strain MBR	99.8
IC-136	*Pseudomonas punonensis* strain LMT03	98.8
IC-117, IC-124, IC-134, IC-135, IC-137, IC-138, IC-140, IC-161-165, IC-49, IC-99	*Pseudomonas resinovorans* strain ATCC 14235	99.3
IC-102	*Pseudomonas songnenensis* strain NEAU-ST5-5	99.4
IC-110, IC-139	*Pseudomonas nitritolerans* strain GL14	99.1
IC-125, IC-126, IC-153, IC-201	*Pseudomonas zhaodongensis* strain NEAU-ST5-21	99.0
IC-250, IC-177, IC-251	*Rhabdobacter roseus* strain R49	99.2
IC-316	*Rhizobium halotolerans* strain AB21	98.6
IC-283	*Rhizobium kunmingense* strain LXD30	99.5
IC-61, IC-86, IC-222, IC-18, IC-21	*Rhizobium rosettiformans* strain W3	99.8
IC-359, IC-364	*Rhodococcus rhodochrous* strain DSM 43241	99.3
IC-297	*Roseomonas aestuarii* strain NBRC 105654	98.9
IC-221	*Roseomonas ludipueritiae* strain 170-96	99.8
IC-306, IC-63, IC-69	*Roseomonas oryzae* strain JC288	99.6
IC-304	*Roseomonas rhizosphaerae* strain YW11	99.4
IC-252	*Roseomonas rosea* strain 173-96	99.1
IC-303	*Sphingobacterium alimentarium* strain WCC 4521	100
IC-262	*Sphingobacterium paludis* strain S37	99.3
IC-330	*Sphingomonas aerolata* strain NW12	99.3
IC-11, IC-25, IC-28, IC-29, IC-55, IC-57, IC-58	*Sphingomonas dokdonensis* strain DS-4	99.1
IC-27, IC-48, IC-229	*Sphingomonas olei* strain K-1-16	99.6
IC-295, IC-287	*Sphingomonas hankookensis* strain ODN7	99.5
IC-208, IC-209, IC-26	*Sphingomonas panaciterrae* strain DCY91	100
IC-231	*Sphingomonas xinjiangensis* strain 10-1-84 1	99.0
IC-104	*Sphingomonas zeae* strain JM-791	99.2
IC-22,30	*Staphylococcus haemolyticus* strain JCM 2416	99.3
IC-242, IC-256, IC-261	*Staphylococcus warneri* strain AW 25	99.5
IC-216, IC-335, IC-337, IC-341, IC-349, IC-350	*Stenotrophomonas rhizophila* strain e-p10	99.5
IC-181, IC-183, IC-184, IC-381, IC-205	*Streptomyces clavifer* strain NRRL B-2557	98.7
IC-207	*Streptomyces camponoticapitis* S3-30	100
IC-198	*Streptomyces niveus* strain NRRL 2466	99.1

Table S8. Activity of antimicrobial iChip isolates from field application against *ESKAPE* pathogens and *Candida albicans*

Isolates	Best BLAST hit	% Identity	***S. aureus***	***E. coli***	***E. facium***	***A. baumannii***	***K. pneumoniae***	***P. aeruginosa***	***C. albicans***
IC-1	*Brevundimonas terrae* strain KSL-145	98.4			O[a]	O	O	O	AO
IC-17	*Brevundimonas terrae* strain KSL-145	98.4				O	O		
IC-18	*Rhizobium rosettiformans* strain W3	98.8	O			O	O		
IC-19	*Massilia varians* strain CCUG 35299	99.5	O						
IC-20	*Brevibacterium frigoritolerans* strain DSM 8801	99.9				O	O		
IC-26	*Sphingomonas panaciterrae* strain DCY91	100			O	O	O	O	AO
IC-52	*Paenibacillus chitinolyticus* strain NBRC 15660	99.5				O	O	O	
IC-73	*Agrobacterium fabrum* strain C58	100	O						
IC-91	*Paenibacillus chitinolyticus* strain NBRC 15660	99.5			O	O	O	O	AO
IC-92	*Paenibacillus chitinolyticus* strain NBRC 15660	99.5	O				O	O	AO
IC-93	*Paenibacillus chitinolyticus* strain NBRC 15660	99.5	O		O		O	O	
IC-130	*Micrococcus yunnanensis* strain YIM 65004	99.7					O		O
IC-181	*Streptomyces clavifer* strain NRRL B-2557	98.7	CE	CE					CE
IC-184	*Streptomyces clavifer* strain NRRL B-2557	98.7	CE						CE
IC-199	*Bacillus nealsonii* strain DSM 15077	99.7		A					
IC-205	*Streptomyces clavifer* strain NRRL B-2557	98.7	CE						CE
IC-207	*Streptomyces camponoticapitis* S3-30	100	CE	CE	CE		CE		
IC-217	*Delftia lacustris* strain 332	99.8				A	A		
IC-249	*Modestobacter versicolor* strain CP153-2	99.5	A						

[a] Antimicrobial activity detected by antagonistic assay (A), soft agar overlay assay (O), both assays (AO), or using crude extracts (CE). Blank cells mean no activity. See Methods for details.

Table S9. See separate excel file.

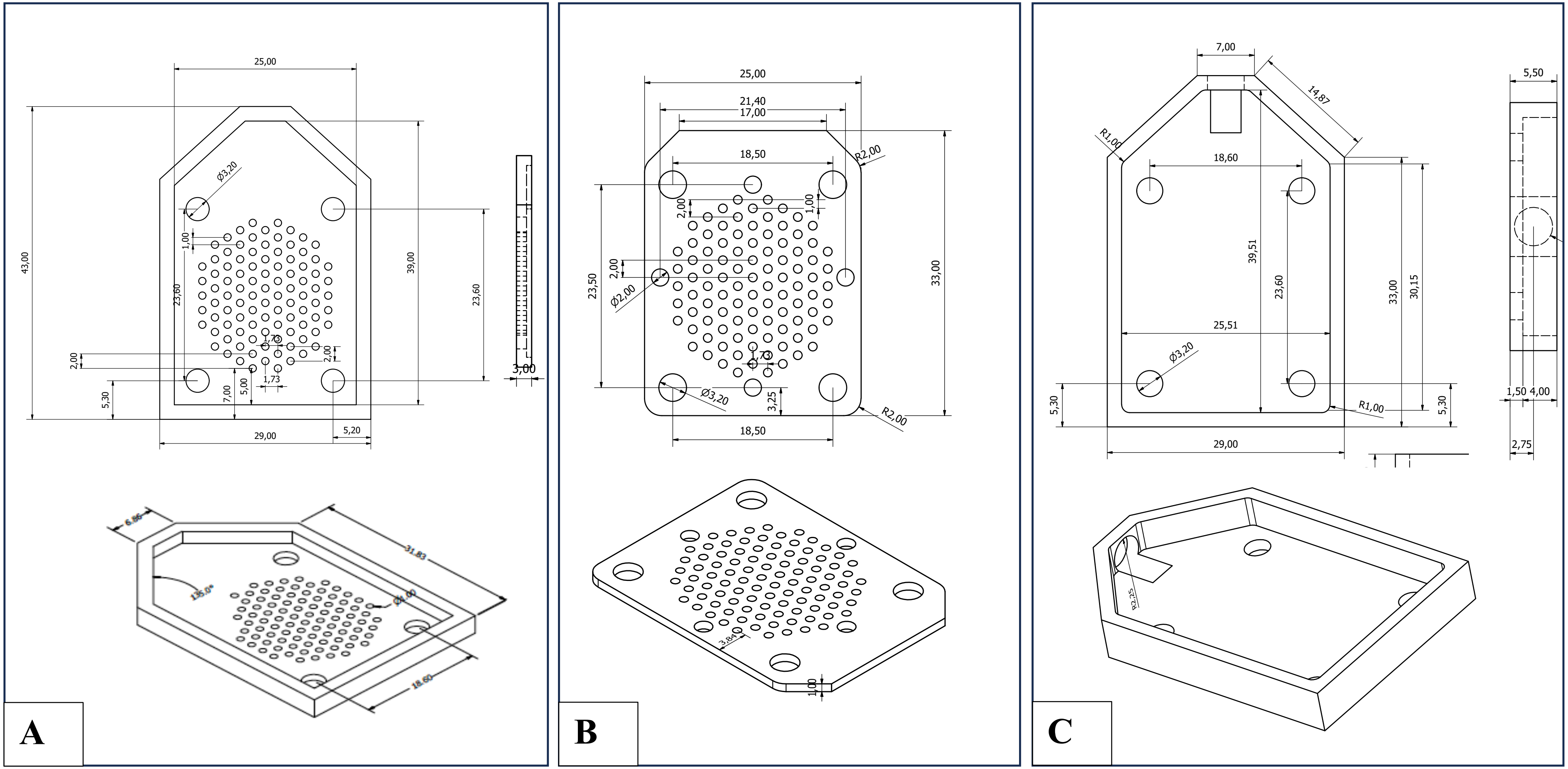

Fig. S1. Schematic drawings of the three iChip components created with Autodesk Inventor.
The images depict the exact dimensions given in mm for the (A) top, (B) central, and (C) bottom compartments of the modified iChip.

B.

A.

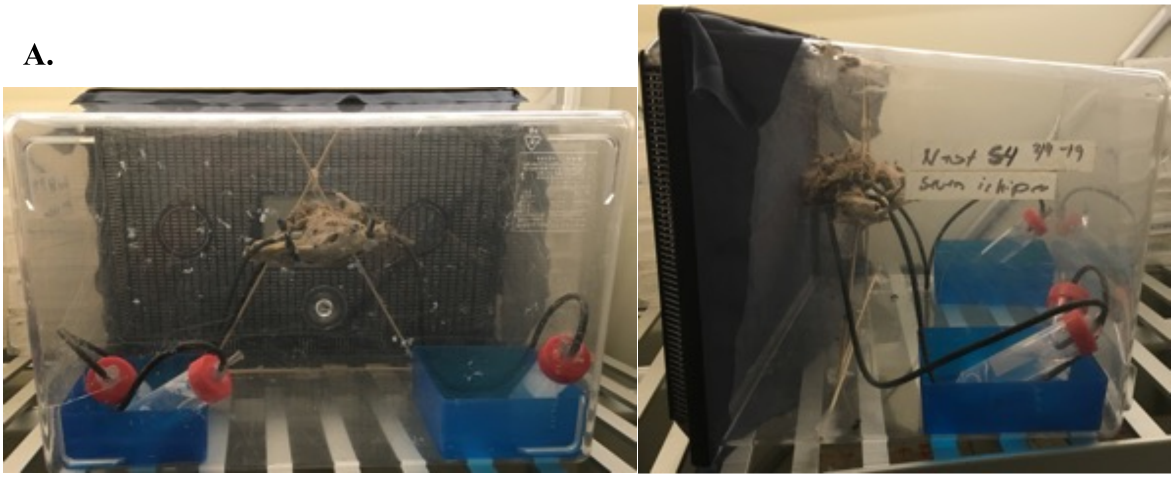

Fig. S2. Photographs of the laboratory setup during *in situ* incubation with the modified iChip. Front (A) and side (B) view of a *S. dumicola* nest with three inserted ichips.

	Total nest microbiome						Cultivation data					
	Nest S2		Nest S3		Nest S4		Nest S2		Nest S3		Nest S4	
	Nest material	Cell extract	Nest material	Cell extract	Nest material	Cell extract	Ichip cultivation	Standard cultivation	Ichip cultivation	Standard cultivation	Ichip cultivation	Standard cultivation
Massilia	15.14	12.49	30.3	8.96	23.5	4.03	0.14	0.063	0.065	0.01	7.84	
Sphingomonas	14.3	11.35	7.1	8.89	7.6	0.66	0.007		0.024	5.94	4.9	6.36
Pseudomonas	0.14	1.11	6	6.91	3.72	12.3	0.13	0.12	0.009	0.01		10
Acinetobacter		0.009	0.16	1.73	0.16	23.18	0.007		0.09	1.98	10.13	
Enterotobacter		1.69		1.61	0.03	21.83	0.054		0.02	3.96	12.09	36.36
Pantoea	1.25	4.15	1.62	2.51	0.92	9.98	0.013		0.003			3.64
Mycoplasma	0.008		6.02	0.006	13.8	0.28						
Planomicrobium	2.98	1.65	0.13	6.54	5.37	0.31				5.9	0.65	
Enterobacteriaceae		0.48	9.02	1.63	0.65	2.21						
Rhizobium	5.02	4.25	1.5	2.27	0.43	0.06		0.009	0.009	0.1		
Novosphingobium	3.64	3.16	1.3	3.39	0.9	0.15						
Uncultured bacterium	3.18	3.28	1.38	2.61	0.61	0.014						
Hymenobacter	1.46	1.14	4.75	0.97	2.04	0.16			0.003	1.98		0.01
Roseomonas	2.81	3.02	1.12	1.9	1.44	0.18	0.054		0.04	3.96	7.52	
Dyadobacter	2.16	4.99	0.19	1.23	1.29	0.091						
Devosia	2.36	1.95	1.37	2.94	1.01	0.029	0.054				0.33	
Adhaeribacter	2.27	1.16	0.79	0.81	3.57							
Methylobacterium	1.72	2.43	1.49	1.64	1.01	0.18	0.09	0.027	0.036	1.98	0.98	
Longimicrobium	2.03	4.62	0.009		0.03			0.009				
Curtobacterium	1.25	2.32	0.68	1.7	0.30	0.15						0.91
Rhodococcus	0.04	0.47	1.55	2.93	1.0	0.06		0.027	0.036	7.92	1.63	3.64
Mucilagnibacter	1.07	2.04	1.35	1.38	0.17							
Pedobacter	0.36	0.32	0.04	0.33	3.85	0.68	0.049		0.009	2.97	4.25	0.91
Chitinophagaceae	2.65	2.22	0.18	0.45	0.05							
Flavisolibacter	2.11	0.02	0.35	2.75	0.11							

Fig. S3. Relative abundance of the 25 most abundant nest microbiome members (amplicons) and their representation in cultivation data. The heat map is sorted according to the sum of the relative abundances for all nests, i.e., the total nest microbiome (amplicons). Grey cells, taxon not detected. Note that relative abundance for amplicons represents 16S rRNA gene abundance, not cell abundance, while for cultivation it refers to the relative number of isolates.

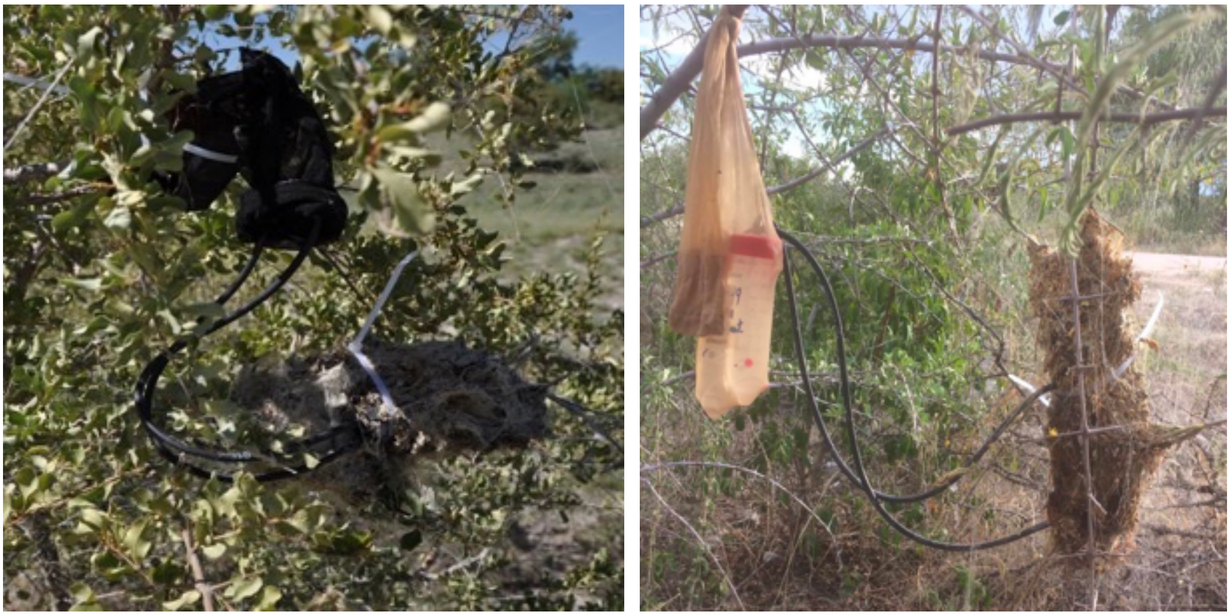

Fig. S4. *In situ* cultivation with the modified iChip during field campaign in Otavi, Namibia. The photographs depict two different *S. dumicola* nests that each contain two iChips connected to water reservoirs situated nearby the nests.

Supplemetary Information: R code used for data analysis

1. **R- script used for trimming, filtering and classification of ASVs.**

```
# This script takes your raw miSeq data (fastq files), trims them (using cutadapt),
# filters and denoises them (using dada2), and classifies the ASVs.
# output: studyName_workspace.RData, which you can load and use for further analysis

# load packages needed
library(cutadapt)  # for trimming your reads
library(dada2)  # for the main analysis

# this will help identify output files later
study <- "Nest_S2" # CHANGE HERE TO MATCH OTHER DATA / SETUP

# get raw read number
setwd("~/Nest_S2_CE_NM//data") # CHANGE HERE TO MATCH OTHER DATA / SETUP
raw.forwards <- sort(list.files(pattern="_R1_001.fastq.gz"))
raw.read.num <- c()
for (filename in raw.forwards) {
  f <- file(filename)
  raw.read.num <- c(raw.read.num, length(readLines(f))/4)
}

# set your working directory

setwd("~/Nest_S2_CE_NM/")  # CHANGE HERE TO MATCH OTHER DATA / SETUP

# get data - unzipped, trimmed data
path <- "./data"  # CHANGE HERE TO MATCH OTHER DATA / SETUP

# trim sequences - CHANGE PRIMER SEQS IF NEEDED
cutadapt_path_PE("data","CCTACGGGNGGCWGCAG","GACTACHVGGGTATCTAATCC")  #
These are the Bac341F and Bac805R MiSeq primers

# Sort ensures forward/reverse reads are in same order
fnFs <- sort(list.files(path, pattern="_R1_001.trimmed.fastq"))
fnRs <- sort(list.files(path, pattern="_R2_001.trimmed.fastq"))
# Extract sample names, assuming filenames have format: SAMPLENAME_XXX.fastq
sample.names <- sapply(strsplit(fnFs, "_"), `[`, 1)
# Specify the full path to the fnFs and fnRs
fnFs <- file.path(path, fnFs)
fnRs <- file.path(path, fnRs)

# visualize quality profiles
plotQualityProfile(fnFs[3:4])
plotQualityProfile(fnRs[3:4])
```

```
# filtering and trimming
filt_path <- file.path(path, "filtered") # Place filtered files in filtered/ subdirectory
filtFs <- file.path(filt_path, paste0(sample.names, "_F_filt.fastq.gz"))
filtRs <- file.path(filt_path, paste0(sample.names, "_R_filt.fastq.gz"))

out <- filterAndTrim(fnFs, filtFs, fnRs, filtRs, truncLen=c(230,230),
              maxN=0, maxEE=c(2,2), truncQ=2, rm.phix=TRUE,
              compress=TRUE, multithread=12) #  # CHANGE HERE TO MATCH OTHER DATA /
SETUP

# learn error rates - NOTE: this takes a long time. especially if you have large samples
errF <- learnErrors(filtFs, multithread=12) # 4 means that we are 4 person using the
processing power of the server
errR <- learnErrors(filtRs, multithread=12)

# visualize error models - remove the hastag if you want to have the plots.
plotErrors(errF, nominalQ=TRUE)
plotErrors(errR, nominalQ=TRUE)

# dereplication - removes all the replicated
derepFs <- derepFastq(filtFs, verbose=TRUE)
derepRs <- derepFastq(filtRs, verbose=TRUE)

# Name the derep-class objects by the sample names
names(derepFs) <- sample.names
names(derepRs) <- sample.names

# infer sequence variants in each sample
dadaFs <- dada(derepFs, err=errF, multithread=12)
dadaRs <- dada(derepRs, err=errR, multithread=12)

# merge paired reads
mergers <- mergePairs(dadaFs, derepFs, dadaRs, derepRs, verbose=TRUE)

# contruct sequence table
seqtab <- makeSequenceTable(mergers)
dim(seqtab)
# Inspect distribution of sequence lengths (of ASVs, not reads!)
lengths <- table(nchar(getSequences(seqtab)))
write.csv(lengths, sprintf("%s_lenghtDistribuiton.csv", study))

# remove chimeras
seqtab.nochim <- removeBimeraDenovo(seqtab, method="consensus", multithread=12,
verbose=TRUE)
dim(seqtab.nochim)
# percentage chimeras
```

```
sum(seqtab.nochim)/sum(seqtab)

# check how many reads made it through the different steps
getN <- function(x) sum(getUniques(x))
track <- cbind(raw.read.num, out, sapply(dadaFs, getN), sapply(mergers, getN),
rowSums(seqtab), rowSums(seqtab.nochim))
# If processing a single sample, remove the sapply calls: e.g. replace sapply(dadaFs, getN)
with getN(dadaFs)
colnames(track) <- c("raw", "trimmed", "filtered", "denoised", "merged", "tabled",
"nonchim")
rownames(track) <- sample.names
track <- as.data.frame(track)
track$percent_survival <- track$nonchim/track$raw
# export so you are sure you can check it later
write.csv2(track, sprintf("%s_SanityCheck.csv", study))

# CLASSIFICATION
# downloaded silva db following tutorial links
(https://zenodo.org/record/824551#.Wh6SVktryL4)

# classify to species level
taxa <- assignTaxonomy(seqtab.nochim,
"~/DBs/SILVA_132_SSURef_Nr99_tax_silva_trunc_dada2.fa.gz", multithread=12)  # CHANGE
HERE TO MATCH OTHER DATA / SETUP
taxa <- addSpecies(taxa, "~/DBs/silva_species_assignment_v132_with3SpeciesDB.fa.gz")  #
CHANGE HERE TO MATCH OTHER DATA / SETUP

# evaluate accuracy
# can only be done if you have mock community

# save R workspace for look at error models + general de-bugging
save.image(file=sprintf("%s_workspace.RData", study))
```

2. **R- script used for decontamination and generation of excel files containing ASV numbers, classification and read numbers.**

```
# This script is a part of the DADA2 16S amplicon analysis pipeline.
# Use on own computer after running DADA_BasicAnalysis.R on a server
# The output from this script is unnormalized data, so you should use
# the next script in line before further data analysis. -> DADA_normalization.R

# things you need to change:
  # working directory - to match with your own computer
  # study name -  must be the same as used in the two previous scripts
  # names of negative controls for the decontamination part

# set your working directory
```

```
setwd("~/Desktop/PhD stuff/Dataanalysis/Amplicon_analysis_New_Ichip/Nest_S2/") #
CHANGE HERE TO MATCH OTHER DATA / SETUP

# install phyloseq - you only have to do this once.
# look at: https://joey711.github.io/phyloseq/install.htmlif you run in to problems
#source('http://bioconductor.org/biocLite.R')
#biocLite('phyloseq')

# load phyloseq
library(phyloseq); packageVersion("phyloseq")

study <- "Nest_S2" # CHANGE HERE TO MATCH OTHER DATA / SETUP

# load workspace data from when you did DADA_BasicAnalysis.R
load(sprintf("%s_workspace.RData", study))

# reset wd
setwd("~/Desktop/PhD stuff/Dataanalysis/Amplicon_analysis_New_Ichip/Nest_S2")   #
CHANGE HERE TO MATCH OTHER DATA / SETUP

# import sample data
samdf <- read.csv("metadata_Nest_S2.csv") # CHANGE HERE TO MATCH OTHER DATA /
SETUP
rownames(samdf) <- samdf[, 1]
samdf[, 1] <- NULL

# make phyloseq object

ps <- phyloseq(otu_table(seqtab.nochim, taxa_are_rows=FALSE),
            sample_data(samdf),
            tax_table(taxa))


plot_richness(ps, x="nest", measures=c("Observed", "Chao1", "Shannon"), color="nest")
ord.nmds.bray <- ordinate(ps, method="NMDS", distance="bray")
plot_ordination(ps, ord.nmds.bray, color="colony", title="Bray NMDS")

top20 <- names(sort(taxa_sums(ps), decreasing=TRUE))[1:20]
ps.top20 <- transform_sample_counts(ps, function(asv) asv/sum(asv))
ps.top20 <- prune_taxa(top20, ps.top20)
plot_bar(ps.top20, x="method", fill="Genus")
+ facet_wrap(~When, scales="free_x")

# get dataframes from phyloseq
data.asv <- as.data.frame(otu_table(ps))
data.tax <- as.data.frame(tax_table(ps))
data.meta <- as.data.frame(sample_data(ps))
```

```
### remove ASVs with short sequences (shorter than 400) and ASVs not classified to Bacteria
nchar(as.character(rownames(data.tax)))
data.tax.longseq <- subset(data.tax, nchar(as.character(rownames(data.tax))) >= 400)
# remove asvs that are not bacteria
data.tax.bact <- data.tax.longseq[data.tax.longseq$Kingdom == "Bacteria", ]

# remove asvs that are chloroplasts or mitochondria
data.tax.bact <- data.tax.bact[data.tax.bact$Order != "Chloroplast", ]
data.tax.bact <- data.tax.bact[data.tax.bact$Family != "Mitochondria", ]
data.tax <- data.tax.bact

data.asv <- data.asv[, colnames(data.asv) %in% rownames(data.tax)]

# replace "NA" with "[higherTaxonomicLevel]"
data.tax <- data.tax[!is.na(data.tax$Kingdom), ] # remove rows where kingdom is NA
data.asv <- data.asv[, colnames(data.asv) %in% rownames(data.tax)]


data.tax <- as.matrix(data.tax)
# manual unUnclassify
for (i in seq(1:nrow(data.tax))) {
  for (j in seq(1:ncol(data.tax))) {
    if (!is.na(data.tax[i, j])){
      mem.tax <- data.tax[i, j]
    } else {
      data.tax[i, j] <- mem.tax
    }
  }
}

data.tax <- as.data.frame(data.tax)

# replace "uncultured" with [higherTaxonomicLevel]"
data.tax <- data.tax[!is.na(data.tax$Kingdom), ] # remove rows where kingdom is NA
data.asv <- data.asv[, colnames(data.asv) %in% rownames(data.tax)]

# manual unUncultured
for (i in seq(1:nrow(data.tax))) {
  for (j in seq(1:ncol(data.tax))) {
    if (!data.tax[i, j] == "uncultured"){
      mem.tax <- data.tax[i, j]
    } else {
      data.tax[i, j] <- mem.tax
    }
  }
```

```
}

data.tax <- as.data.frame(data.tax)

# make ASV overview df
colnames(data.asv) == rownames(data.tax)  # check that things line up
numbers <- paste("ASV_", seq(1:length(colnames(data.asv))), sep="")  # make list of ASV_1
numbers
ASVs <- cbind(numbers, colnames(data.asv), data.tax)  # make df with seqs + tax
rownames(ASVs) <- ASVs$numbers
colnames(ASVs)[2] <- "seq"

# exchange seqs for numbers in data tax and data asv
# this makes the dataframes much faster to import, and makes them more readable in excel
colnames(data.asv) <- numbers
rownames(data.tax) <- numbers

# checking that everything matches up
rownames(data.meta) == rownames(data.asv)
rownames(data.tax) == colnames(data.asv)

# filter away non-bact
data.tax.bact <- data.tax[data.tax$Kingdom == "Bacteria", ]
data.asv.bact <- data.asv[, colnames(data.asv) %in% rownames(data.tax.bact)]

# reset variables
data.tax <- data.tax.bact
data.asv <- data.asv.bact
data.meta <- data.meta
data.meta$sampleID <- rownames(data.meta)

################### DECONTAMINATION
##############################################
neg_control_samples <- c("MRH40") # CHANGE HERE TO MATCH OTHER DATA / SETUP
EXACT NAME OF THE NEGATIVE CONTROLS
neg_control_samples <- rownames(data.meta[data.meta$nest == "neg", ]) # you can use
this line if your negative controls has "neg" written in some metadata category
# how many more times can a ASV be present in a real sample than in neg ctrl, and still be
suspect.
# e.g. 0.2: has to be 5x more in real sample to be OK
# adjust this based on you data, and on how stringent you want to be
multiplication_factor <- 0.2

check_negative_controls <- function(data.asv, neg_control_samples, multiplication_factor){
  sample_names_no_negatives <- row.names(data.asv)[row.names(data.asv) %in%
neg_control_samples == FALSE]
  suspect_asvs = c()
```

```
  for(i in 1:dim(data.asv)[2]){
    sample_read_counts <- data.asv[sample_names_no_negatives,i]
    negative_read_counts <- data.asv[neg_control_samples,i]
    if(max(negative_read_counts) > multiplication_factor*max(sample_read_counts)){
      suspect_asvs[length(suspect_asvs)+1] <- colnames(data.asv)[i]
    }
  }
  return(suspect_asvs)
}

suspect_asvs <- check_negative_controls(data.asv, neg_control_samples,
multiplication_factor)

# consider the suspect ASVs
data.asv.contaminants <-  data.asv[, colnames(data.asv) %in% suspect_asvs]
data.tax.contaminants <- data.tax[rownames(data.tax) %in% suspect_asvs, ]
data.contaminants <- cbind(data.tax.contaminants,
as.data.frame(t(data.asv.contaminants)))
# look at data contaminants to see which ASVs you are about to remove from the dataset

# ...and remove them from your data if you want to
data.asv <- data.asv[, !colnames(data.asv) %in% suspect_asvs]
data.tax <- data.tax[!rownames(data.tax) %in% suspect_asvs, ]

# remove neg.ctrl sample from data
data.meta <- data.meta[!rownames(data.meta) %in% neg_control_samples, ]
data.asv <- data.asv[!rownames(data.asv) %in% neg_control_samples, ]

################### EXPORT CONTAMINATION INFO
##################################
# export non-normaized data - only bact
write.csv2(data.contaminants, sprintf("%s_contaminantsRemoved_TAXandASVtable.csv",
study))

################### EXPORT UNNORMALIZED DATA
###################################

# export dataframes
write.csv2(ASVs, sprintf("%s_DADA_seqTAXtable.csv", study))
write.csv2(data.asv, sprintf("%s_DADA_ASVtable.csv", study))
write.csv2(data.tax, sprintf("%s_DADA_TAXtable.csv", study))
write.csv2(data.meta, sprintf("%s_DADA_SAMPLEtable.csv", study))

# also make tax.asv table for easy overview
data.asv.t <- as.data.frame(t(data.asv))
colnames(data.asv.t) == (rownames(data.meta))
colnames(data.asv.t) <- paste(colnames(data.asv.t), data.meta$nest)
```

```
data.tax.asv <- cbind(data.tax, data.asv.t)
write.csv2(data.tax.asv,  sprintf("%s_DADA_TAX_and_ASVtable.csv", study))
```

3. R-script used for normalization.

```
# This script is a part of the DADA2 16S amplicon analysis pipeline.
# Previous script in normal workflow: DADA_phyloseq_filter_decontamination.R

# things you need to change:
  # working directory - to match with your own computer
  # study name - must be the same as used in the two previous scripts

# set your working directory
setwd("~/Desktop/PhD stuff/Dataanalysis/Amplicon_analysis_New_Ichip/Nest_S2/") #
CHANGE HERE TO MATCH OTHER DATA / SETUP

# set study name
study <- "Nest_S2" # CHANGE HERE TO MATCH OTHER DATA / SETUP

# import raw data
data.asv <- read.csv2(sprintf("%s_DADA_ASVtable.csv", study), row.names=1)
data.tax <- read.csv2(sprintf("%s_DADA_TAXtable.csv", study), row.names=1)
data.meta <- read.csv2(sprintf("%s_DADA_SAMPLEtable.csv", study), row.names=1)

################### FRACTION OF TOTAL READS
#######################################

# set a minimun number of reads e.g. 1st Quartile of the asv data summed per sample
#same as above
#min_reads <- summary(rowSums(data.asv))[2] # Calculate 1st Quartile
min_reads <- 1
# transform asv table to fit with old code
data.asv.t <- as.data.frame(t(data.asv))
# discard samples with less than you minimum of reads reads
data.asv.t.minreads <- data.asv.t[, which(colSums(data.asv.t) >= min_reads)]
data.meta.norm <- data.meta[which(rownames(data.meta) %in%
colnames(data.asv.t.minreads)), ]

# fractionify

data.asv.t.norm <- data.asv.t.minreads
for (i in 1:ncol(data.asv.t.norm)){
  k <- sum(data.asv.t.norm[, i])
  for (j in 1:nrow(data.asv.t.norm)){
    data.asv.t.norm[j,i] <- data.asv.t.norm[j,i]/k
  }
```

```
}

# just checking that everything sums to 1
colSums(data.asv.t.norm)


# make tax-asv overview table
rownames(data.tax) == rownames(data.asv.t.norm)
data.tax.asv.norm <- cbind(data.tax, round(data.asv.t.norm,))
sample_nest_names <- paste(data.meta.norm$nest, data.meta.norm$sampleID)
colnames(data.tax.asv.norm)[8:ncol(data.tax.asv.norm)] <- sample_nest_names

# export
write.csv(data.asv.t.norm, sprintf("%s_DADA_Fraction_ASVtable.csv", study))
write.csv(data.tax, sprintf("%s_DADA_Fraction_TAXtable.csv", study))
write.csv(data.meta.norm, sprintf("%s_DADA_Fraction_SAMPLEtable.csv", study))
write.csv(data.tax.asv.norm, sprintf("%s_DADA_Fraction_TAXandASVtable.csv", study))
```

4. R-script used to sum ASVs to Genus level.

```
################# IMPORT ###############################################
data.asv.norm <- read.csv(sprintf("%s_DADA_Fraction_ASVtable.csv", study), row.names=1)
data.tax.norm <- read.csv(sprintf("%s_DADA_Fraction_TAXtable.csv", study), row.names=1)
data.meta.norm <- read.csv(sprintf("%s_DADA_Fraction_SAMPLEtable.csv", study),
row.names=1)


#choose the level you wish to sum data on
tax.level <- "Genus"
# insert column in asv data
data.asv.norm$tax.level <- data.tax.norm[, tax.level]

data.asv.norm <- na.omit(data.asv.norm)

library(dplyr) # this package is needed for summation

Summation <- data.asv.norm <- as.data.frame(data.asv.norm %>%
                              group_by(tax.level) %>%
                              summarise_all(list(~sum(.))))


write.csv(Summation,'Genus_summed_Nest_S2.csv', sep = ";")
```

5. **R-script used to create heatmaps.**

```
data <- read.csv("FigureS3.csv",sep = ",") #Opload data
rnames <- data[,1] # Remove rownames and construct an numeric dataframe
mat_data <- data.matrix(data[,2:ncol(data)])
rownames(mat_data) <- rnames # Add rownames

library(reshape2)
library(ggplot2)
library(scales)


# melt data into long format

melt_data <- melt(data)

ggplot(melt_data, aes(variable, Genus)) + # x and y axes => Var1 and Var2
  geom_tile(aes(fill = value)) + # background colours are mapped according to the value
column
  geom_text(aes(fill = melt_data$value, label = round(melt_data$value, 2))) + # write the
values indside the cells
  scale_fill_gradient(low = "white", high = "red", na.value = "grey") +
  theme(axis.title.x = element_blank()) +# removing x-axis label
  theme(axis.text.y = element_text(angle = 360, hjust = 1, size = 10, color = 'black')) + #
changes to y-axis text
  theme(strip.text = element_text(size=12)) +
  theme(legend.text=element_text(size=12)) +
  theme(panel.spacing= unit(0.1, "cm")) +
  ylab("Genus")  # adding y-axis label
```
